# Machine learning in vascular surgery: a systematic review and critical appraisal

**DOI:** 10.1038/s41746-021-00552-y

**Published:** 2022-01-19

**Authors:** Ben Li, Tiam Feridooni, Cesar Cuen-Ojeda, Teruko Kishibe, Charles de Mestral, Muhammad Mamdani, Mohammed Al-Omran

**Affiliations:** 1 Department of Surgery, University of Toronto, 149 College St, Toronto, ON M5T 1P5 Canada; 2 Division of Vascular Surgery, St. Michael’s Hospital, Unity Health Toronto, 30 Bond Street, Toronto, ON M5B 1W8 Canada; 3 Temerty Centre for Artificial Intelligence Research and Education in Medicine (T-CAIREM), University of Toronto, 1 King’s College Circle, Toronto, ON M5S 1A8 Canada; 4 Health Sciences Library, St. Michael’s Hospital, Unity Health Toronto, 209 Victoria St, Toronto, ON M5B 1T8 Canada; 5 Li Ka Shing Knowledge Institute, St. Michael’s Hospital, Unity Health Toronto, 209 Victoria St, Toronto, ON M5B 1T8 Canada; 6 Institute of Health Policy, Management and Evaluation, Dalla Lana School of Public Health, University of Toronto, 155 College St, Toronto, ON M5T 3M7 Canada; 7 Leslie Dan Faculty of Pharmacy, University of Toronto, 144 College St, Toronto, ON M5S 3M2 Canada; 8 Institute of Medical Science, University of Toronto, 1 King’s College Circle, Toronto, ON M5S 1A8 Canada; 9 Department of Surgery, King Saud University, ZIP 4545, Riyadh, 11451 Kingdom of Saudi Arabia

**Keywords:** Vascular diseases, Health care

## Abstract

Machine learning (ML) is a rapidly advancing field with increasing utility in health care. We conducted a systematic review and critical appraisal of ML applications in vascular surgery. MEDLINE, Embase, and Cochrane CENTRAL were searched from inception to March 1, 2021. Study screening, data extraction, and quality assessment were performed by two independent reviewers, with a third author resolving discrepancies. All original studies reporting ML applications in vascular surgery were included. Publication trends, disease conditions, methodologies, and outcomes were summarized. Critical appraisal was conducted using the PROBAST risk-of-bias and TRIPOD reporting adherence tools. We included 212 studies from a pool of 2235 unique articles. ML techniques were used for diagnosis, prognosis, and image segmentation in carotid stenosis, aortic aneurysm/dissection, peripheral artery disease, diabetic foot ulcer, venous disease, and renal artery stenosis. The number of publications on ML in vascular surgery increased from 1 (1991–1996) to 118 (2016–2021). Most studies were retrospective and single center, with no randomized controlled trials. The median area under the receiver operating characteristic curve (AUROC) was 0.88 (range 0.61–1.00), with 79.5% [62/78] studies reporting AUROC ≥ 0.80. Out of 22 studies comparing ML techniques to existing prediction tools, clinicians, or traditional regression models, 20 performed better and 2 performed similarly. Overall, 94.8% (201/212) studies had high risk-of-bias and adherence to reporting standards was poor with a rate of 41.4%. Despite improvements over time, study quality and reporting remain inadequate. Future studies should consider standardized tools such as PROBAST and TRIPOD to improve study quality and clinical applicability.

## Introduction

Machine learning (ML) is a rapidly advancing field of artificial intelligence (AI) that enables computer technology to learn from data to identify patterns and make predictions without explicit programming^1^. The field has been driven by the explosion of electronic data combined with increasing computational power^2^. ML techniques are increasingly applied to solve health care problems, with its global market value predicted to grow from $4.9 billion in 2020 to $45.2 billion by 2026^3^. The value of ML/AI is that these technologies can automatically and quickly analyze large amounts of data to augment a clinician’s ability to diagnose disease and make predictions about outcomes, among other applications^4^. Compared to traditional statistical techniques, ML applies advanced computing technology to more accurately model complex relationships in large datasets^5^.

Vascular surgery is highly suitable for ML applications for several reasons. First, the endovascular revolution has made vascular surgery a field that is oriented toward technology and medical imaging, facilitating the application of powerful ML-based image analysis software^6,7^. Second, there are objective clinical definitions for most vascular conditions (e.g., abdominal aortic aneurysm [AAA] defined as size ≥3 cm^8^ and peripheral artery disease [PAD] defined as ankle brachial index <0.9^9^). This allows ML algorithms to automate diagnosis with little human input^10^. Third, vascular surgical procedures are often high-risk and performed on patients with multiple comorbidities^11^. Therefore, it is critical to make accurate predictions about post-operative outcomes using previous experience, which ML is designed for^12^. Finally, there is a growing abundance of data available to facilitate the development of ML models through the Vascular Quality Initiative, which captures patient-level data across 796 centers in North America^13^.

ML algorithms have been applied to predict AAA growth^14^, detect endoleaks^15^, and identify patients with PAD who have high mortality risk^16^. Despite an increasing amount of research interest in ML techniques, its translation to real-world practice remains limited. One reason for this could be inadequate quality or reporting of existing studies, reducing clinical applicability. Several standardized tools, including the Prediction Model Risk of Bias Assessment (PROBAST)^17^ and Transparent Reporting of a Multivariable Prediction Model for Individual Prognosis or Diagnosis (TRIPOD)^18^, have been developed to assess the risk-of-bias and adherence to reporting standards for prediction models. Surveys of physicians demonstrate that significant barriers to the adoption of AI/ML technologies are lack of knowledge and trust in these models^19,20^. The application of standardized quality assessment tools such as PROBAST and TRIPOD can provide clinicians with more effective mechanisms to evaluate AI/ML tools and determine applicability to their practice^21,22^.

Given recent advances in ML technology and its potential to transform clinical practice, it is important to understand its applications to vascular surgical conditions. Systematic reviews have been conducted on ML/AI in neurosurgery^23^, plastic surgery^24^, and orthopedic surgery^25^. However, there has been no synthesis or evaluation of ML studies in vascular surgery using standardized tools such as PROBAST and TRIPOD. We conducted a systematic review and critical appraisal to comprehensively synthesize and rigorously evaluate the ML literature in vascular surgery.

## Results

### Study screening and selection

**Fig. 1:**
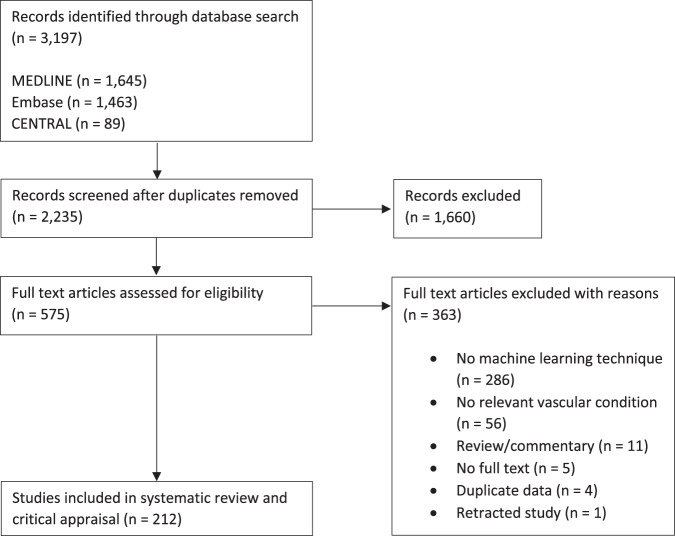
PRISMA study flow diagram. Summary of number of articles screened and included.

We identified 3197 articles through our search of MEDLINE (*n* = 1645), Embase (*n* = 1463), and Cochrane CENTRAL (*n* = 89). A total of 2235 articles remained after duplicates were removed, all of which underwent title and abstract screening. A total of 1660 records were excluded and 575 underwent full-text review. A total of 363 were excluded, most commonly because there was no ML technique (*n* = 286) or relevant vascular condition (*n* = 56). Hand-search of reference lists identified no additional articles. In all, 212 studies were included in the final systematic review and critical appraisal. Our search results are summarized in the Preferred Reporting Items for Systematic Reviews and Meta-Analyses (PRISMA) study flow diagram (Fig. 1).

### Study characteristics

**Fig. 2:**
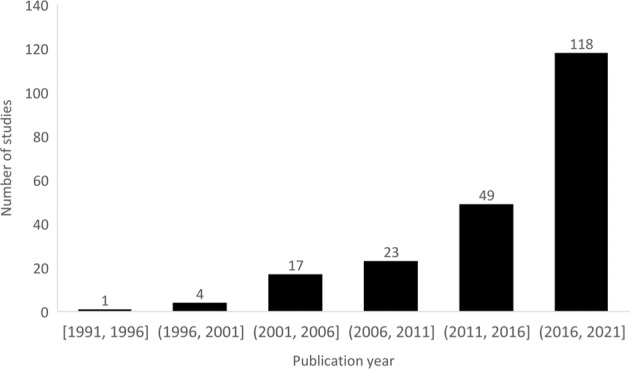
Publications trends for machine learning studies in vascular surgery between 1991 and 2021. Each bar represents a 5-year interval.

**Fig. 3:**
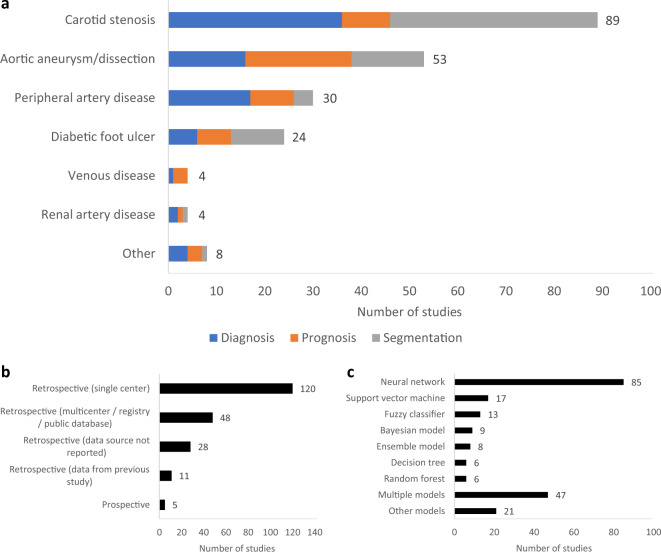
Characteristics of included studies. **a** Disease conditions and objectives, **b** study design, and **c** machine learning models applied.

We included 212 studies published between 1991 and 2021. The number of publications increased significantly from 1 (1991–1996) to 118 (2016–2021) (Fig. 2). Articles reported on carotid stenosis (*n* = 89), aortic aneurysm/dissection (*n* = 53), PAD (*n* = 30), diabetic foot ulcer (*n* = 24), venous disease (*n* = 4), renal artery disease (*n* = 4), and other vascular conditions (*n* = 8). The main goals of the studies were diagnosis (*n* = 82), prognosis (*n* = 55), and image segmentation (*n* = 75) (Fig. 3a). Most studies were published in the US (*n* = 56), China (*n* = 37), and UK (*n* = 19). A summary of included studies is presented in Supplementary Table 1.

### Design, populations, and follow-up

Most studies developed their model using retrospective, single-center data (*n* = 120) and many did not report the data source (*n* = 28). Only five studies were prospectively tested, and no randomized controlled trials were conducted (Fig. 3b). Median sample size was 170 patients (range 1–1,567,636) and 141/212 (66.5%) studies had ≥100 patients. The median event rate was 48.7% (range 0.6–85.6%) and 69/104 (66.3%) of studies had an event rate >30%. Of note, 44/55 (80%) prognostic studies did not report the length of follow-up.

### Machine learning methods

The most commonly applied ML model was a neural network (*n* = 85), particularly convolutional neural network (*n* = 42). Other ML models included support vector machine (*n* = 17), fuzzy classifier (*n* = 13), Bayesian model (*n* = 9), ensemble model (*n* = 8), decision tree (*n* = 6), and random forest (*n* = 6). A significant proportion of studies applied multiple ML models (*n* = 47) (Fig. 3c).

Most studies used imaging/doppler signals as the sole input feature (*n* = 150). A total of 41 studies used structured clinical/demographic/laboratory/genomic data alone, 16 used both structured and imaging data, 3 used clinical notes alone, and 2 used both structured variables and clinical notes as predictors.

Of the studies that reported a validation method, k-fold cross-validation was the most common (*n* = 80) followed by leave-one-out cross-validation (*n* = 17), while bootstrapping was less common (*n* = 3). External validation was performed in 9/212 (4.2%) studies.

### Aortic aneurysm and dissection

Diagnostic studies focused on detection of aortic aneurysm/dissection, rupture, and endoleak (*n* = 16). Prognostic studies included prediction models for aneurysm growth/rupture and mortality/re-intervention after surgery (*n* = 16). Input features were imaging alone (*n* = 13), structured clinical variables alone (*n* = 13), or a combination of structured and imaging data (*n* = 6). Image segmentation algorithms were designed to identify aortic true/false lumens and thrombus on computed tomography (CT) (*n* = 13). Sample sizes ranged from 8 to 1,049,160 with a median of 143 patients. Event rates ranged from 1.5 to 71.4% with a median of 38.4%. The area under the receiver operating characteristic curve (AUROC) ranged from 0.61 to 0.99 with a median of 0.87.

### Carotid stenosis

Diagnostic studies were focused on detecting the presence/degree of carotid stenosis (*n* = 22) and classification into symptomatic vs. asymptomatic status (*n* = 14). Prognostic studies included prediction of stenosis progression/stroke risk (*n* = 3), shunt necessity during endarterectomy (*n* = 1), and cardiovascular events following revascularization (*n* = 2). Input features were imaging/doppler signals alone (*n* = 29), structured data alone (*n* = 8), and a combination of structured and imaging features (*n* = 5). Most image segmentation algorithms were designed to identify carotid intima/media and plaque on ultrasound (*n* = 31). Several magnetic resonance imaging-based studies segmented carotid plaque to identify high-risk features such as ulceration, intraplaque hemorrhage, and necrotic core (*n* = 5). Sample sizes ranged from 10 to 90,000 with a median of 161 patients. Event rates ranged from 3.6 to 76.3% with a median of 52.6%. AUROC ranged from 0.75 to 0.99 with a median of 0.90.

### Peripheral artery disease

Diagnostic studies were focused on detecting the presence/severity of PAD (*n* = 10) and differentiating ischemic vs. neurogenic claudication (*n* = 2). Several ML models for patients with lower extremity prostheses were designed to detect falls and determine terrain type (*n* = 2). Prognostic studies included prediction of mortality/complications and health care utilization in patients with PAD (*n* = 4), ambulation potential after amputation (*n* = 3), and surgical site infection following lower extremity bypass (*n* = 1). Input features included imaging/functional data alone such as CT, ultrasound, and walking motion data (*n* = 13), structured clinical information (*n* = 9), and a combination of structured and imaging data (*n* = 1). Two studies used clinical notes to identify patients with PAD using natural language processing. Sample sizes ranged from 1 to 253,125 with a median of 265 patients. Event rates ranged from 12.3 to 70.0% with a median of 23.1%. AUROC ranged from 0.61 to 1.00 with a median of 0.89.

### Diabetic foot ulcer

Diagnostic studies were focused on detection of ulceration (*n* = 1), infection/ischemia (*n* = 1), and neuropathy (*n* = 2) using pictures and plantar pressures. Prognostic studies predicted risk of amputation (*n* = 2), mortality (*n* = 2), and ulcer healing (*n* = 1) based on structured clinical variables and imaging data. Image segmentation studies localized ulcers based on pictures (*n* = 6) and thermograms (*n* = 2). Two studies more specifically segmented ulcers into granulation, necrotic, or slough tissue from pictures. Sample sizes ranged from 5 to 1,567,636 with a median of 207 patients. Event rates ranged from 0.6 to 85.6% with a median of 41.2%. AUROC ranged from 0.71 to 1.00 with a median of 0.84.

### Venous disease

ML techniques were applied to venous disease through detection of venous thromboembolism (*n* = 1) and prediction of varicose vein development (*n* = 1) and venous ulcer development/healing (*n* = 2). Two studies used structured clinical data alone as input features, and two used a combination of structured and imaging data. Sample sizes ranged from 77 to 493,519 with a median of 325 patients. Event rates ranged from 1.9 to 51.9% with a median of 50.0%. AUROC ranged from 0.70 to 0.86 with a median of 0.78.

### Renal artery stenosis

ML techniques were applied for detection of renal artery stenosis from completion angiogram (*n* = 1) and captopril renography (*n* = 1). One study applied ML technology to identify relationships between covariates and outcomes in the Cardiovascular Outcomes in Renal Atherosclerotic Lesions trial^26^. Sample sizes ranged from 29 to 573 with a median of 150 patients. The median event rate was 21.2%. AUROC ranged from 0.68 to 0.93 with a median of 0.81.

### Other vascular conditions

ML studies on other vascular conditions included detection and prediction of vascular injury in anterior lumbar spine surgery using clinical data and operative notes (*n* = 1), prediction of cardiovascular mortality/re-admission after major vascular surgery (*n* = 2), identification of arteriovenous fistula stenosis (*n* = 1), detection of lymphedema (*n* = 1), and endovascular guidewire tracking (*n* = 2). Sample sizes ranged from 30 to 246,205 with a median of 78 patients. The event rate ranged from 7.2 to 46.2%, with a median of 30.3%. AUROC ranged from 0.68 to 0.92, with a median of 0.81.

### Outcomes

**Fig. 4:**
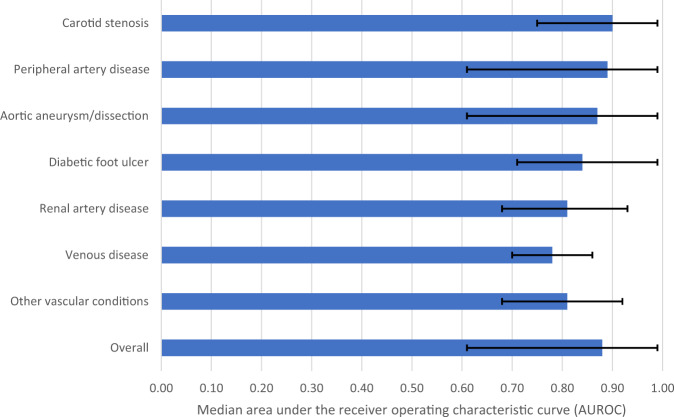
Median area under the receiver operating characteristic curve (AUROC) across included studies by disease condition. Black bars represent ranges.

The main outcome measures to assess the performance of ML models were AUROC, sensitivity, specificity, and accuracy. The ranges and proportion of studies with values ≥80% were the following: AUROC (0.61–1.00; 62/78 [79.5%] studies ≥80%), sensitivity (30–100%, 62/77 [80.5%] studies ≥80%), specificity (52–100%, 64/75 [85.3%] studies ≥80%), accuracy (67–100%, 100/109 [91.7%] studies ≥80%). Median AUROC across included studies was 0.88 (range 0.61–1.00) and a summary of AUROC’s (medians and ranges) across each disease condition is presented in Fig. 4.

Twenty-two studies compared the outcomes of their ML model to clinicians, existing risk prediction tools, or traditional regression models. Twenty performed better, two performed similarly, and none performed worse. Specifically, 6 performed better than traditional regression models such as logistic, linear, and Cox regression^16,27–31^, 11 performed better than existing risk prediction tools such as the Glasgow Aneurysm Score, Mangled Extremity Severity Score (MESS), and Padua Prediction Score^32–42^, 1 performed better than vascular surgeons in predicting in-hospital mortality following AAA repair^43^, and 2 performed better than radiologists in detecting AAA on CT^15,44^. One performed similarly to logistic regression for predicting shunt necessity during carotid endarterectomy^45^ and another demonstrated no difference compared to radiologists in detecting aortic dissection on CT^46^. A summary of these findings can be found in the outcomes column of Supplementary Table 1.

### Risk-of-bias assessment

**Fig. 5:**
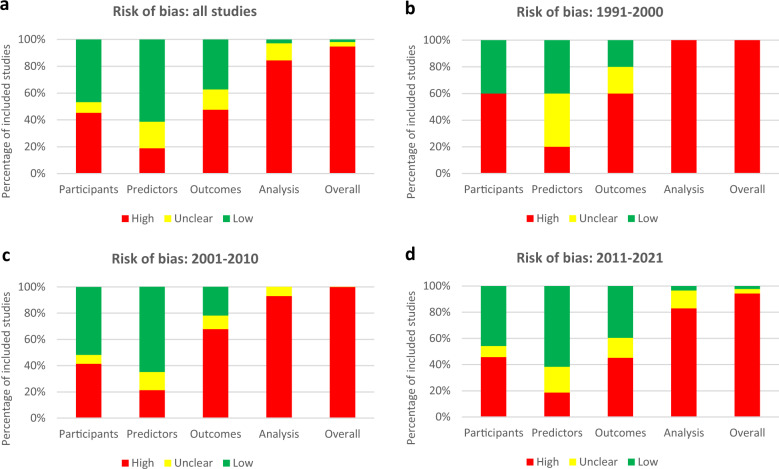
Risk-of-bias assessment of included studies using Prediction Model Risk of Bias Assessment Tool (PROBAST). **a** All studies and **b** studies published between 1991 and 2000, **c** 2001 and 2010, and **d** 2011 and 2021.

Of the 212 included studies, overall risk-of-bias was high for 201 (94.8%), unclear for 7 (3.3%), and low for 4 (1.9%). High risk in the analysis domain (179/212 [84.4%] studies) was the main contributor to a study being overall high risk. Specifically, many studies did not report the number of participants with missing data, perform calibration to assess model performance, or account for overfitting. In the participants' domain, 96/212 (45.3%) were high risk mainly because inclusion and exclusion criteria for their study cohort were not described. Similarly, in the outcomes domain, 101/212 (47.6%) were high risk because outcomes were not defined, blinding was not performed, and the time interval between predictor assessment and outcome determination was not reported. In the predictors' domain, some studies were high/unclear risk (82/212 [38.7%]) due to inadequate definition of predictors and unclear availability of predictor data at the time of model application. The proportion of low risk-of-bias studies increased in each domain between publication years 1991–2000 and 2011–2021: participants (40.0% vs. 45.8%), predictors (40.0% vs. 61.6%), outcomes (20.0% vs. 39.5%), and analysis (0% vs. 3.4%). Study quality improved over time, with overall low risk-of-bias studies published only after 2010 (Fig. 5a–d). There were four studies judged to be at low risk-of-bias: Perkins (2020)^40^, Ross (2016)^16^, Ravaut (2021)^47^, and Ross (2019)^48^.

### High-quality studies

Perkins (2020) used US registry data to develop a Naïve Bayes model that predicted the risk of amputation following lower extremity revascularization and externally validated the algorithm on a UK registry with an AUROC of 0.97^40^. The authors demonstrated that their ML model performed better than the existing MESS^40^. Ross (2016) applied decision trees to detect PAD and predict mortality using a combination of clinical, imaging, and genomic data with better predictive ability than logistic regression^16^. Ravaut (2021) developed an ML model from 1,567,636 patients using over 700 clinical variables from administrative health data to predict diabetes complications including amputations with an AUROC of 0.78^47^. Ross (2019) generated a prediction model from 7,686 patients using 1000 variables that were readily available from electronic health records including clinical data and notes to predict major adverse cardiovascular events in patients with PAD with an AUROC of 0.81^48^. These four studies appropriately defined their study population, predictors, and outcomes, as well as reported discrimination performance, model calibration, and supplementary data describing how readers can apply the models to their own practice.

### Adherence to reporting standards

**Fig. 6:**
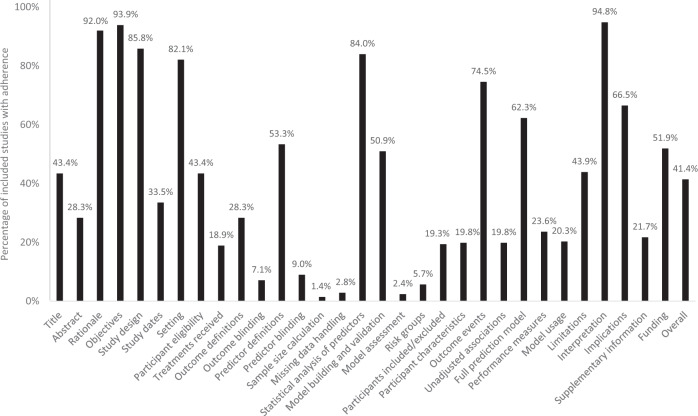
Reporting adherence of included studies to Transparent Reporting of a Multivariable Prediction Model for Individual Prognosis or Diagnosis (TRIPOD) tool. Proportion of articles with adherence to each TRIPOD category is represented.

**Fig. 7:**
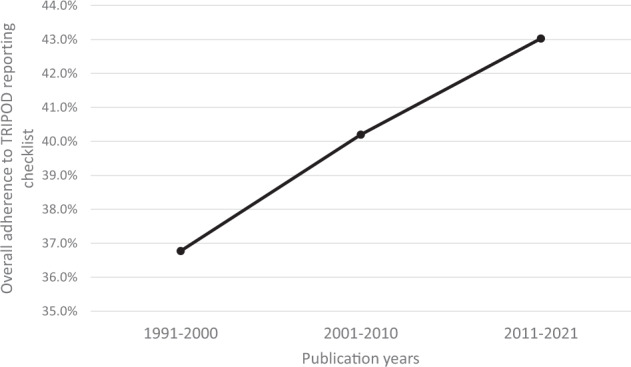
Time trend for overall adherence to TRIPOD tool based on publication year between 1991 and 2021. Ten-year intervals are represented.

Overall adherence to the TRIPOD reporting checklist was 41.4%, with 19/31 domains having a rate less than 50% (Fig. 6). Reporting adherence was above 90% for study rationale, objectives, and interpretation, but below 10% for blinding of outcomes/predictors, sample size calculation, missing data handling, model assessment, and identification of risk groups. In particular, less than 20% of studies adequately defined their study population in terms of inclusion/exclusion criteria and baseline characteristics. Furthermore, less than 30% of abstracts reported sufficient information regarding study methodology and about 50% of studies did not disclose funding sources. Concerningly, fewer than one in four studies provided information on how their ML model could be used by readers. Overall adherence to TRIPOD items improved over time based on publication year: 1991–2000 (36.8%), 2001–2010 (40.2%), 2011–2021 (43.0%) (Fig. 7).

## Discussion

### Summary of findings

This systematic review and critical appraisal of 212 studies published over 30 years provides a comprehensive synthesis and rigorous evaluation of the ML literature in vascular surgery. The research interest in ML has grown significantly, with a substantial increase in the number of publications between 1991 and 2021. However, some vascular conditions remain understudied, such as venous disease and renal artery stenosis, which account for less than 5% of publications. Convolutional neural networks were the most commonly applied model, reflecting the preference for advanced technology^49^. Current ML algorithms have excellent predictive ability, with a median AUROC of 0.88. Disease conditions with more publications had higher median AUROC’s (carotid stenosis [0.90], aortic aneurysm/dissection [0.87], PAD [0.89]), while those with fewer publications had lower median AUROC’s (diabetic foot ulcer [0.84], renal artery stenosis [0.81], venous disease [0.78]). Several ML models performed better than existing clinical prediction tools (*n* = 11), clinicians (*n* = 3), and traditional regression models (*n* = 6). However, overall risk-of-bias was high in 94.8% of studies and adherence to reporting standards was suboptimal at 41.8%. Most studies developed ML models using retrospective, single-center data and did not report the length of follow-up. External validation was performed in less than 5% of studies. Despite improvements over time, study quality and reporting remain poor.

### Comparison to the existing literature

One systematic review of AI in AAA was published in 2020, identifying 34 studies that used AI for image segmentation, diagnosis, and prognosis^50^. However, the study did not capture ML techniques in PAD, carotid stenosis, diabetic foot ulcers, venous disease, and other vascular conditions. Furthermore, quality assessment of included studies was not performed. Our systematic review captured a broader spectrum of vascular conditions and applied standardized tools (PROBAST and TRIPOD) to critically evaluate the ML literature in vascular surgery.

The predictive potential of ML has been demonstrated in other surgical specialities. For example, Senders et al. (2018) conducted a systematic review of 34 publications on ML models for outcome prediction in neurosurgery^51^. The authors demonstrated a median accuracy and AUROC of 94.5% and 0.83, respectively^51^. Their ML algorithms had 15% greater accuracy than logistic regression and most performed better than existing prognostic indices and clinicians^51^. Our study similarly demonstrated excellent predictive outcomes for ML algorithms in vascular surgery. The median AUROC of ML models in our studies was 0.88, with 6 performing better than traditional regression techniques, 11 performing better than existing risk prediction tools, and 3 performing better than clinicians. For example, Ross et al. (2016) developed an ML model with an AUROC that was 0.11 higher than logistic regression for PAD detection and mortality prediction^16^. Perkins et al. (2020) showed that their ML algorithm performed better than the MESS at predicting outcomes following lower extremity revascularization for trauma patients (AUROC 0.97 vs. 0.74)^40^. Talebi et al. (2020) compared their ML model to generalist radiologists for the detection of AAA from CT and demonstrated an accuracy that was 5–25% higher^15^.

Previous groups have analyzed the risk-of-bias and adherence to reporting standards for ML studies. Nagendran et al. (2020) conducted a systematic review evaluating the outcomes of deep learning prediction models versus clinicians^52^. They demonstrated that overall risk-of-bias was high in 58/81 (71.6%) studies based on PROBAST criteria and there was <50% adherence to 12 TRIPOD items^52^. Similarly, Wynants et al. (2020) assessed 169 prediction models for COVID-19 diagnosis/prognosis and determined that overall risk-of bias was high or unclear for all of them^53^. We also demonstrated a high risk-of-bias and poor adherence to reporting standards for ML studies in vascular surgery.

### Implications

ML has gained tremendous interest in recent years but remains a relatively novel field, particularly with respect to health care applications^54^. Most studies on ML models in vascular surgery have been published in the past 5 years. Furthermore, standardized guidelines on the conduct of ML studies have not been widely adopted^55^. These reasons likely explain the suboptimal quality and reporting of current studies. We demonstrate improvements in study quality and adherence to reporting standards over time, suggesting that higher research quality coincides with the development of the field. Furthermore, our data suggest that model performance improves with the increasing application of ML techniques to vascular surgery, particularly in aortic, carotid, and PAD. We also identified disease conditions that require greater attention including diabetic foot ulcers, venous disease, and renal artery stenosis.

ML has significant advantages over traditional risk prediction tools as they can learn from a wide range of data types, including structured clinical, laboratory, and genetic information along with unstructured imaging data and clinical notes^56^. However, few ML studies in vascular surgery have leveraged this technological advantage, with many using solely structured or unstructured data as predictors. Ross et al. (2019) used a combination of unstructured text data from clinical notes and structured information from diagnostic/procedural codes, prescriptions, vital signs, and laboratory investigations to predict major adverse cardiac and cerebrovascular events in PAD patients^48^. The authors performed a sensitivity analysis demonstrating that the removal of text data decreased model performance from an AUROC of 0.81–0.78 (*p* = 0.002)^48^. Following the example set by Ross et al.^48^, future studies should consider training ML models on multiple data types to potentially increase predictive power.

A distinguishing feature of ML models is their ability to learn continuously to improve performance^57^. However, most studies did not describe how the reader could apply their algorithm nor provide source code. This makes it challenging to test the model in different clinical settings and build on existing algorithms. To improve clinical applicability and accelerate the advancement of the field, future studies should consider publishing their de-identified raw data and source codes through repositories such as GitHub^58^.

Given the novelty of the field, most ML studies have been developed and tested on retrospective data. There are currently no randomized controlled trials assessing the impact of this technology on vascular surgical outcomes. Furthermore, few studies externally validated their algorithm. It will be critical for future studies to assess the impact of ML models on clinically relevant outcomes and their ability to function in different clinical settings. For example, Perkins et al. (2020) developed an ML algorithm to predict outcomes following lower extremity revascularization in trauma patients using data from the US Joint Trauma System and externally validated their model on the UK Joint Theatre Trauma Registry^40^. The authors demonstrated that their algorithm maintained excellent performance in their external validation population, with an AUROC of 0.97^40^. Perkins et al. then developed an internationally accessible website for clinicians to apply their model (https://www.traumamodels.com/). Future work on ML in vascular surgery should follow this example in developing generalizable, accessible, and clinically relevant tools.

It is also essential for ML models to consider biases including gender, racial, and socioeconomic disparities^59^. Less than 20% of studies in our systematic review reported inclusion/exclusion criteria and demographic characteristics for their study population. This poses a significant risk of prediction tools disadvantaging minority populations. Future work should ensure that their study cohort captures an appropriately diverse population.

Perkins (2020)^40^, Ross (2016)^16^, Ravaut (2021)^47^, and Ross (2019)^48^ are four studies judged to be at low risk-of-bias with good potential for broad clinical implementation in vascular surgery. These papers provided a detailed description of their study population with inclusion and exclusion criteria, reported specific definitions for their variables and outcomes of interest, identified the specific timepoint during a patient’s clinical course when their algorithm should be applied, and assessed model performance using various calibration methods^16,40,47,48^. Future work should look toward these publications for guidance on study methodology and consider building on their algorithms.

Developing and implementing successful ML models in vascular surgery requires a detailed and systematic approach, which has been described by others^60–63^. Generally, the first consideration is devising a specific, clinically relevant question with input from end-users^60^. Then, it is critical to build a team of clinicians, computer scientists, and administrators with expertise in patient care and model development^60^. Together, this group can assess whether there is sufficient quantity and quality of data available to develop a model that can adequately address the problem of interest^60^. Given that overfitting can be a significant problem, it is recommended to create simple models without an abundance of extraneous features that do not contribute to predictive performance^61^. A multidisciplinary team can provide guidance on selecting important input variables to inform an accurate model^61^. Furthermore, evaluating the generalizability of the algorithm and its associated biases is essential prior to clinical implementation^62^. Importantly, the model’s impact on patient outcomes and clinician workflow should be prospectively evaluated, particularly in vascular surgery where patients often undergo high-risk, urgent interventions^62^. Finally, post-implementation evaluation with regular performance monitoring and system retraining with up-to-date information is important given the constant evolution of clinical practice and datasets^62^.

### Limitations

This study has several limitations. PROBAST and TRIPOD are designed to assess prediction models, but have not been validated specifically for ML applications. However, previous studies have used PROBAST and TRIPOD to evaluate ML models as quality assessment criteria for clinical prediction tools and ML algorithms are similar^22,52,53^. Currently, work is underway to develop a TRIPOD-ML tool^64^. Furthermore, there may be publication bias, with high-performing ML models being more likely to be published.

## Conclusions

Our systematic review and critical appraisal of 212 studies demonstrates that ML models have excellent predictive power in vascular surgery within the research setting with a median AUROC of 0.88. Many models performed better than traditional regression techniques, existing prediction tools, and clinicians. ML technology can provide powerful augmentation to clinicians for image analysis, disease diagnosis, and outcome prediction. However, risk-of-bias and adherence to reporting guidelines are currently substandard, likely due to the novelty of the field. Given the need for ML algorithms to be rigorously validated prior to clinical implementation, future studies should strongly consider standardized tools such as PROBAST and TRIPOD to guide study design and reporting.

## Methods

### Protocol and registration

A systematic review was conducted according to the PRISMA statement guidelines^65,66^. Our study protocol (CRD42021240310) was registered with the International Prospective Register of Systematic Reviews^67^. Ethics approval was not required for this study as this was a systematic review of published articles.

### Information sources and search strategy

Our search strategy was devised in consultation with an experienced librarian (TK). MEDLINE, Embase, and the Cochrane Central Register of Controlled Trials (CENTRAL) were searched from inception to March 1, 2021, for studies reporting ML applications in vascular surgery. A combination of Medical Subject Heading terms, keywords, and synonyms for ML AND vascular surgery were used to maximize sensitivity. EndNote Version 20 was used to collate references^68^. We hand-searched the reference lists of included studies for additional relevant articles. Our search did not apply language limitations and Google Translate was used for non-English studies^69^. The search strategy is detailed in Supplementary Table 2.

### Study selection and data collection

Title and abstract screening, full-text review, data collection, and assessment of risk-of-bias and reporting adherence were conducted by two independent reviewers (BL and TF), with a third author resolving discrepancies (CC-O). Covidence was used to facilitate the systematic review^70^. We included all original studies reporting ML applications in vascular surgery, including case reports, case series, observational studies, and clinical trials. Reviews, commentaries/editorials/letters, animal studies, and articles without full text were excluded.

A standardized form was used to collect data for included studies based on the Critical Appraisal and Data Extraction for Systematic Reviews of Prediction Modelling Studies Checklist^71^. Variables obtained were study authors, publication year, country, data collection period, disease condition, study objective (i.e., diagnosis/prognosis/image segmentation), study design, data source, ML model, input features, prediction outputs, sample size, training/validation/test sets, validation method, reporting of external validation, follow-up, and outcomes. Authors were contacted through email for relevant information not reported in the original publication.

### Data analysis and critical appraisal

Publications trends were assessed by plotting the number of included studies in 5-year intervals between the first and last published articles (1991–2021). Bar graphs were developed to summarize the number of papers focused on the different vascular conditions, main goal (diagnosis/prognosis/segmentation), ML model applied, and study design. Study outcomes including AUROC, sensitivity, specificity, and accuracy were summarized as medians and/or ranges across included articles and percentage of studies reporting values ≥80%. This threshold represents the excellent discriminatory ability of a prediction model^72^.

Critical appraisal was performed by assessing the risk-of-bias and adherence to reporting standards for individual articles and collating the results to determine the overall quality of included studies. Specifically, risk-of-bias was assessed using the PROBAST^17^. PROBAST assesses four domains (participants, predictors, outcomes, and analysis) with 20 study methodology questions to determine overall risk-of-bias^17^. Reporting adherence was assessed using the TRIPOD tool^18^. TRIPOD is a 31-item checklist that provides reporting standards for prediction model studies^18^. Trends over time for included studies based on PROBAST risk-of-bias and TRIPOD adherence was assessed in 10-year intervals based on publication year (1991–2000, 2001–2010, and 2011–2021).

All numerical analyses were conducted using R version 4.0.3 (R Project for Statistical Computing).

## Supplementary information

Supplementary Information

## Data Availability

All relevant data are available through the paper and supplement. Additional information is available from the authors upon reasonable request.

## Code availability

The code used in this study is available from the authors upon reasonable request.
