# Supplementary material for: Machine learning in vascular surgery: a systematic review and critical appraisal
Source: NPJ Digit Med. 2022 Jan 19;5:7. doi: 10.1038/s41746-021-00552-y (PMC8770468; doi:10.1038/s41746-021-00552-y)
Supplement: Supplementary file 1 — Supplementary Information [file 41746_2021_552_MOESM1_ESM.pdf]

Supplementary Information

Supplementary Table 1. Summary of Included Studies

Author/ Year	Country	Design	Data source	Machine learning model	Input features	Prediction outputs	Sample size	Event rate	Validation method	Outcomes
**Abdominal aortic aneurysm**										
**Diagnosis**										
Shum 2011[1]	US	Retro	Single institution	Decision tree	CT	Detection of AAA rupture	76	13.2%	10-fold cross-validation	Ac 86.6%
Macia 2011[2]	Spain	Retro	NR	Neural network (multilayer perceptron)	CT	Detection of endoleak	NR	NR	10-fold cross-validation	Sn 94.4%, Sp 92.9%, Ac 93.7%
Zhang 2013[3]	US	Retro	Single institution	Ensemble (Adaboost)	CT	Detection of AAA rupture	60	50.0%	NR	AUROC 0.87, Ac 91.7%
Garcia 2014[4]	Spain	Retro	Single institution	Multiple (k-nearest neighbour, neural network, SVM)	CT	Detection of endotension	36	47.2%	10-fold cross-validation	AUROC 0.96, Sn 95.2%, Sp 91.5%, Ac 94.4%
Li 2018[5]	US	Retro	3 institutions	Other (regularized linear model)	14 clinical variables and genome sequencing	Detection of AAA	474	66.0%	10-fold cross-validation	AUROC 0.80
Madani 2019[6]	US	Retro	Single institution	CNN (U-Net)	CT	Detection of endoleak	70	71.4%	NR	AUROC 0.89, Ac 82.4%, precision 81.4%, recall 83.6%, better compared to 2 blinded radiologists: Ac 85-89%, precision 77-98%, recall 78-99%
Kerut 2019[7]	US	Retro	Single institution	Neural network (feed-forward)	18 demographic/clinical variables	Detection of AAA	10,329	2.7%	NR	Mean square error 0.025
Mommers 2020[8]	Netherlands	Retro	3 institutions	ANN	Detection of volatile organic compounds in exhaled air	Detection of AAA (screening)	101	34.7%	Leave-10%-out cross-validation	AUROC 0.84, Sn 83.0%, Sp 81.0%
Talebi 2020[9]	US	Retro	Single institution	CNN (U-Net)	CT	Detection of endoleak	70	71.4%	NR	AUROC 0.99, Ac 95.0%, precision 90.0%, recall 100%, better than generalist radiologists (Ac 70-90%, precision 50-83%, recall 70-100%), similar to cardiovascular subspecialist (Ac 100%, precision 100%, recall 100%)
McLenon 2021[10]	US	Retro	Registry (statewide)	Other (machine learning)	Imaging study reports from EMR	Detection of AAA	230,660	NR	NR	Absence of AAA (Sn 91.9%, PPV 77.7%), presence of AAA (Sn 88.5%, PPV 95%)
**Prognosis**										
Turton 2000[11]	UK	Retro	Single institution	Neural network	4 peri-operative variables	Prediction of 30-day mortality following open ruptured AAA repair	102	52.9%	NR	Sn 86.4%, Sp 79.3%, Ac 82.5%, PPV 82.6%, NPV 88.5%
Hadjianastassiou 2006[12]	UK	Retro	24 institutions	ANN	4 clinical variables	Prediction of in-hospital mortality following AAA repair	1,751	31.2%	10-fold cross-validation	AUROC 0.87, better compared to clinicians 0.82
Lee 2013[13]	US	Retro	3 institutions	Multiple (Decision tree, Naïve Bayes, k-nearest neighbour, SVM)	CT	Prediction of emergency AAA repair	205	45.4%	10-fold cross-validation	Ac 85.5%, better than maximum aneurysm diameter alone 68.9%

Author/ Year	Country	Design	Data source	Machine learning model	Input features	Prediction outputs	Sample size	Event rate	Validation method	Outcomes
Attallah 2014[14]	UK	Retro	2 datasets from 2 institutions	Bayesian neural network	Peri-operative clinical and imaging characteristics	Prediction of 5-year re-intervention after EVAR	146	43.8%	10-fold cross-validation	AUROC 0.81
Wise 2015[15]	US	Retro	Single institution	ANN	Pre-operative variables	Prediction of in-hospital mortality following ruptured AAA repair	125	42.4%	4-fold cross-validation	AUROC 0.88, better than logistic regression 0.85 and Glasgow Aneurysm Score 0.77
Karthikesalingam 2015[16]	UK	Retro	2 institutional prospective databases	ANN	Demographic/clinical variables and pre-operative CT characteristics	Prediction of long-term mortality and complications following EVAR	761	NR	NR	NR
Monsalve-Torra 2016[17]	Spain	Retro	Single institution	Multiple (multilayer perceptron, radial basis function, Bayesian network)	57 peri-operative variables	Prediction of in-hospital mortality following open AAA repair	317	7.4%	10-fold cross-validation	Sn 87.0%, Sp 96.1%, Ac 95.4%
Thompson 2016[18]	US	Retro	Single institution	ANN	Peri-operative characteristics	Prediction of mortality in patients with ruptured AAA	64	29.7%	NR	Poor agreement with other risk predictors (Glasgow Aneurysm Score [K = 0.14], Vancouver Score [K = 0.46], Edinburgh Ruptured Aneurysm Score [K = 0.25], VSGNE rAAA Risk Score [K = 0.79])
Attallah 2017[19]	UK	Retro	2 datasets from 2 institutions	Multiple (ANN, SVM, K-nearest neighbours)	45 morphological variables from CT	Prediction of re-intervention following EVAR	743	8.9%	5-fold cross-validation	Sn 87.0%
Attallah 2017[20]	UK	Retro	2 institutions	ANN	47 peri-operative/imaging variables	Prediction of 5-year re-intervention following EVAR	743	8.9%	5-fold cross-validation	AUROC 0.61, Sn 73.0%
Lee 2018[21]	UK	Retro	Prospective database	SVM	AAA and brachial artery diameter	Prediction of AAA growth	94	NR	Nested 5-fold cross-validation	AUROC 0.80, Ac 85.0%
Parikh 2018[22]	US	Retro	Single institution	Decision tree	CT	Prediction of emergency AAA repair	150	50.0%	NR	Ac 81.0%
Canchi 2018[23]	Singapore	Retro	Single institution	Multiple (Decision tree, Naïve Bayes, SVM)	9 clinical variables and 8 CT characteristics	Prediction of AAA rupture	312	8.3%	10-fold cross-validation	AUROC 0.97, Ac 95.2%
Meng 2019[24]	US	Retro	Drug database	Bayesian confidence propagation neural network	Antibiotic usage	Prediction of aortic aneurysm/dissection development	3,721	NR	NR	NR
Hirata 2020[25]	Japan	Retro	Single institution	Ensemble (XGBoost)	9 features from CT	Prediction of AAA expansion > 4mm/year	50	24.0%	10-fold cross-validation	AUROC 0.86
Ding 2020[26]	China	Retro	Single institution	Neural network (multilayer perceptron)	CT	Prediction of AAA progression following EVAR	99	38.4%	10-fold cross validation	AUROC 0.90, Ac 85.2%
Zhou 2020[27]	China	Retro	2 institutions	Multiple (SVM, random forest)	Clinical notes from EMR	Prediction of acute kidney injury and paraplegia after thoracoabdominal aortic aneurysm repair	212	12.7%	5-fold cross-validation	AUROC 0.89
Hyer 2020[28]	US	Retro	National registry	Other (stochastic hill-climbing)	Demographic/clinical variables	Prediction of 30- and 90-day mortality, readmission, and complications following AAA repair	1,049,160	4.4%	NR	AUROC 0.87, better than Charlson Comorbidity Index (0.72), Elixhauser Comorbidity Index (0.80), and Centers for Medicare and Medicaid Service's Hierarchical Condition Category (0.86)

Author/ Year	Country	Design	Data source	Machine learning model	Input features	Prediction outputs	Sample size	Event rate	Validation method	Outcomes
Mei 2020[29]	US	Retro	National registry (Medicare)	CNN	Peri-operative variables	Prediction of 30-day and long-term mortality following EVAR vs. open AAA repair	7,826	50.6%	NR	EVAR survival 83.5 days compared to 79.2 days for open repair
Kordzadeh 2020[30]	UK	Prosp	Single institution	Multiple (ANN, decision tree, naïve Bayes, SVM)	26 pre-operative variables	Prediction of endoleak following EVAR	241	29.0%	5-fold cross-validation	Ac 86.0%
Jalalahmadi 2020[31]	US	Retro	Single institution	Decision tree (J48)	15 clinical variables and 30 CT characteristics	Prediction of AAA rupture	66	NR	5-fold cross-validation	AUROC 0.75, Sn 75.0%, Sp 86.0%, Ac 82.0%
Rengarajan 2020[32]	US	Retro	Single institution	Multiple (Naïve Bayes, k-nearest neighbour, decision tree, random forest, SVM, Lasso)	Clinical variables and CT characteristics	Prediction of symptomatic AAA	150	33.3%	Leave-one-out cross-validation	AUROC 0.89, Sn 78.0%, Sp 92.0%, Ac 87.0%
**Image segmentation**										
Olabarriaga 2005[33]	Netherlands	Retro	Single institution	Other (k-nearest neighbours)	CT	Segmentation of aortic thrombus	17	N/A	NR	Volume overlap 95%, volume error 4.5%, mean segmentation error 1.3mm, segmentation time 24.5s
Shum 2010[34]	US	Retro	Single institution	Neural network	CT	Segmentation of AAA lumen, outer wall, inner wall, and measurement of wall thickness	20	50.0%	NR	Correlation coefficient 0.98 (ruptured), 1.0 (non-ruptured), mean error 3.69%
Maiora 2014[35]	Spain	Retro	NR	Random forest	CT	Segmentation of AAA	8	N/A	NR	NR
Maiora 2014[36]	Spain	Retro	NR	Random forest	CT	Segmentation of AAA thrombus	8	N/A	NR	Ac 98.0%
Jordanski 2018[37]	US	Retro	Single institution	Multiple (multivariate linear regression, multilayer perceptron neural network)	Vascular models	Segmentation of AAA and carotid artery based on wall shear stress	4,000	N/A	5-fold cross-validation	Gaussian conditional random fields coefficient of determination 0.95
Wang 2018[38]	China	Retro	Single institution	CNN	CT/MRI	Segmentation of aorta into lumen, thrombus, outer wall, and calcification	21	N/A	NR	Ac (CT: 99.1%, MRI: 98.5%)
Lopez-Linares 2018[39]	Spain	Retro	Single institution	CNN	CT	Segmentation of aortic thrombus following EVAR	13	N/A	4-fold cross-validation	Dice coefficient 82.0%
Mohammadi 2019[40]	Iran	Retro	Single institution	CNN	CT	Segmentation of AAA lumen, outer wall, body border, and bone	5,800	N/A	5-fold cross validation	Ac 98.6%
Chandrashekar 2020[41]	UK	Retro	Single institution	CNN (modified U-Net)	CT	Segmentation of AAA lumen and wall structure	75	N/A	3-fold cross-validation	Ac (contrast enhanced CT: 93.4%, non-contrast CT: 88.7%)
Fantazzini 2020[42]	Italy	Retro	Single institution	CNN (U-Net)	CT	Segmentation of aortic lumen	80	N/A	NR	Dice coefficient 0.92, segmentation time 25s
Caradu 2020[43]	France	Retro	Single institution	CNN	CT	Segmentation of aortic lumen and thrombus	100	N/A	NR	Sn 94.0%, Sp 97.0%, Pearson's correlation coefficient 0.90, Dice coefficient 0.95
He 2021[44]	France	Retro	Single institution	Multiple (neural network (multilayer perceptron), random forest, LASSO)	Aneurysm tissue samples	Segmentation of thoracic aortic aneurysm wall stress	15	N/A	NR	Mean square error 0.16, R2 = 0.91

Author/ Year	Country	Design	Data source	Machine learning model	Input features	Prediction outputs	Sample size	Event rate	Validation method	Outcomes
**Aortic dissection**										
**Diagnosis**										
Harris 2019[45]	US	Retro	Single institution	CNN	CT	Detection of aortic dissection or rupture	778	64.1%	NR	Dissection (Sn 87.8%, Sp 96.0%), rupture (Sn 100.0%, Sp 96.0%), median reduction time of 395s between study intake and radiologists review
Huo 2019[46]	China	Retro	Single institution	Bayesian network	All EMR variables	Detection of aortic dissection	492	67.1%	10-fold cross-validation	AUROC 0.86, precision 84.6%
Cheng 2020[47]	China	Retro	Single institution	CNN (U-Net)	CT	Detection of aortic dissection	20	50.0%	5-fold cross-validation	Sn 90.0%, Sp 80.0%, Ac 85.0%
Liu 2020[48]	China	Retro	Single institution	Ensemble (AdaBoost, SmoteBagging, EasyEnsemble)	76 clinical/laboratory variables from EMR	Detection of aortic dissection	60,000	1.7%	7-fold cross validation	Sp 79.2%, recall 78.1%
Liu 2020[49]	China	Retro	Single institution	Ensemble (AdaBoost, XGBoost, SmoteBagging, EasyEnsemble)	42 demographic/clinical/laboratory variables from EMR	Detection of aortic dissection (screening)	53,213	1.5%	7-fold cross validation	Sn 80.5%, Sp 79.5%, computation time 117s
Hata 2021[50]	Japan	Retro	Single institution	CNN (Xception architecture)	CT	Detection of aortic dissection	170	55.9%	5-fold cross-validation	AUROC 0.94, Sn 91.8%, Sp 88.2%, Ac 90.0%, no difference in performance compared to radiologists
**Image segmentation**										
Cao 2019[51]	China	Retro	Single institution	CNN	CT (pre-operative)	Segmentation of aorta, true lumen, and false lumen in aortic dissection	276	N/A	3-fold cross-validation	Dice coefficient: 0.93 (aorta), 0.93 (true lumen), 0.91 (false lumen), segmentation speed 0.038s/image
Chen 2021[52]	China	Retro	Multiple institutions	CNN	CT (pre-operative)	Segmentation of true lumen, false lumen, and branch vessels in aortic dissection	120	N/A	6-fold cross validation	Dice coefficient true lumen (0.94), false lumen (0.91), run time 0.91s
Yu 2021[53]	China	Retro	Single institution	CNN	CT	Segmentation of true and false lumen in aortic dissection	139	N/A	NR	Dice coefficient 0.96 (aorta), 0.96 (true lumen), 0.93 (false lumen), r = 0.99, measurement time 21.7 min, measurement time faster than manual segmentation 82.5 min
**Carotid stenosis**										
**Diagnosis**										
Serhatlioglu 2003[54]	Turkey	Retro	NR	Neural network (feed-forward)	Carotid artery doppler signals	Classification of degree of carotid stenosis (low/moderate/high)	118	NR	NR	Ac 85.0%
Christodoulou 2003[55]	UK	Retro	Single institution	Multiple (neural network, k-nearest neighbour)	U/S	Classification of symptomatic vs. asymptomatic carotid plaque	230	50.0%	Five bootstrap sets	AUROC 0.75, Ac 73.1%
Yyldyrym 2004[56]	Turkey	Retro	Single institution	Neural network (multilayer perceptron, radial basis function)	U/S	Classification of degree of carotid stenosis (normal/low/moderate/severe)	80	50.0%	NR	Ac 0.86, Sn 86.7%, Sp 90.0%, Ac 87.5%
Mofidi 2005[57]	Ireland	Retro	Single institution	ANN	Duplex velocity measurements	Classification of degree of carotid stenosis (10% intervals)	208	54.8%	NR	Sn 97.3%, Sp 97.7%, Ac 97.5%, $R2$ = 0.94, more accurate than duplex velocity criteria (discriminant power 4.11 vs 1.67, $p < 0.05$)

Author/ Year	Country	Design	Data source	Machine learning model	Input features	Prediction outputs	Sample size	Event rate	Validation method	Outcomes
Kyriacou 2005[58]	UK	Retro	Single institution	Multiple (k-nearest neighbours, neural network, SVM)	U/S	Classification of symptomatic vs. asymptomatic carotid plaque	274	50.0%	Leave-one-out cross-validation	Ac 67.0%
Kyriacou 2005[59]	UK	Retro	2 datasets from 2 institutions	Multiple (k-nearest neighbours, neural network, SVM)	U/S	Classification of symptomatic vs. asymptomatic carotid plaque	610	55.1%	Leave-one-out cross-validation	Ac 71.2%
Ubeyli 2005[60]	Turkey	Retro	NR	Neural network (multilayer perceptron)	Carotid artery doppler signals	Detection of carotid stenosis	160	70.0%	Cross-validation on 12 patients	Ac 96.0%
Ubeyli 2005[61]	Turkey	Retro	NR	Other (neuro-fuzzy system)	Carotid artery doppler signals	Detection of carotid stenosis	130	68.5%	NR	Ac 96.6%
Ubeyli 2005[62]	Turkey	Retro	Single institution	Neural network (multilayer perceptron)	Carotid artery doppler signals	Detection of carotid stenosis	160	70.0%	NR	Sn 97.4%, Sp 96.4%, Ac 97.0%
Guler 2005[63]	Turkey	Retro	NR	Neural network (multilayer perceptron)	Carotid artery doppler signals	Detection of carotid stenosis	160	70.0%	NR	Sn 97.4%, Sp 96.4%, Ac 97.0%
Ubeyli 2006[64]	Turkey	Retro	Single institution	Neural network (multilayer perceptron)	Carotid artery doppler signals	Detection of carotid stenosis	130	68.5%	NR	Sn 96.2%, Sp 96.0%, Ac 96.2%
Mougiakakou 2007[65]	UK	Retro	Single institution	ANN	U/S	Classification of symptomatic vs. asymptomatic carotid plaque	108	50.0%	NR	AUROC 0.92
Ozsen 2007[66]	Turkey	Retro	NR	Multiple (Supervised Affinity Maturation, ANN, decision tree)	Carotid artery doppler signals	Detection of carotid stenosis	114	52.6%	10-fold cross validation	Sn 99.6%, Sp 97.7%, Ac 98.9%
Polat 2007[67]	Turkey	Retro	Single institution	Multiple (Fuzzy clustering and SVM)	Carotid artery doppler signals	Detection of carotid stenosis	114	52.6%	10-fold cross validation	Sn 100%, Sp 100%, Ac 100%
Ceylan 2008[68]	Turkey	Retro	Single institution	ANN (wavelet transformed-complex-valued)	Carotid artery doppler signals	Detection of carotid stenosis	78	48.7%	2-fold cross-validation	Sn 100%, Sp 100%, Ac 100%
Ubeyli 2008[69]	Turkey	Retro	NR	Neural network (probabilistic)	Carotid artery doppler signals	Detection of carotid stenosis	50	68.0%	NR	Sn 97.6%, Sp 96.9%, Ac 98.2%
Ubeyli 2008[70]	Turkey	Retro	NR	Neural network (multilayer perceptron)	Carotid artery doppler signals	Detection of carotid stenosis	160	70.0%	NR	Sn 100%, Sp 96.9%, Ac 98.2%
Acharya 2011[71]	US	Retro	NR	SVM	U/S	Classification of symptomatic vs. asymptomatic carotid plaque	160	31.3%	3-fold cross-validation	AUROC 0.89, Sn 97.0%, Sp 80.0%, Ac 91.7%
Acharya 2012[72]	UK	Retro	Single institution	Ensemble (AdaBoost)	U/S	Classification of carotid plaque into symptomatic vs. asymptomatic	346	56.6%	3-fold cross-validation	AUROC 0.82, Sn 82.9%, Sp 82.1%, Ac 82.4%
Acharya 2012[73]	Italy	Retro	Single institution	Multiple (SVM, k-nearest neighbours, radial basis probabilistic neural network)	U/S	Classification of symptomatic vs. asymptomatic carotid plaque	346	56.6%	k-fold cross-validation	Sn 89.6%, Sp 88.9%, Ac 89.5%, PPV 99.7%
Uguz 2012[74]	Turkey	Retro	Single institution	Multiple (neural network, SVM, k-nearest neighbours, Naïve-bayes)	Carotid artery doppler signals	Detection of carotid stenosis	191	71.2%	5-cross fold validation	AUROC 0.97, Sn 98.5%, Sp 96.4%, Ac 97.9%
Acharya 2013[75]	Italy	Retro	Single institution	SVM	CT	Classification of symptomatic vs.	20	55.0%	10-fold cross-validation	Sn 90.2%, Sp 86.5%, Ac 88.0%, PPV 85.5%

Author/ Year	Country	Design	Data source	Machine learning model	Input features	Prediction outputs	Sample size	Event rate	Validation method	Outcomes
						asymptomatic carotid plaque				
Acharya 2013[76]	Portugal, UK	Retro	2 datasets from 2 institutions	Multiple (fuzzy classifier, SVM, neural network, decision tree, k-nearest neighbour, Bayes classifier)	U/S	Classification of symptomatic vs. asymptomatic carotid plaque	492	48.8%	3-fold cross-validation	Dataset 1 (Sn 99.0%, Sp 80.0%, Ac 93.1%, PPV: 91.8%), Dataset 2 (Sn 84.4%, Sp 85.9%, Ac 85.3%, PPV 82.3%)
Gastounioti 2015[77]	Greece	Retro	2 institutions	SVM	U/S	Classification of symptomatic vs. asymptomatic carotid plaque	56	50.0%	NR	Ac 88.0%
Bhosale 2018[78]	Finland	Retro	Population-based study	Other (Lasso penalized logistic regression)	Serum protein biomarkers	Detection of carotid stenosis	86	50.0%	3-fold cross-validation	AUROC 0.79
Molinari 2018[79]	Italy	Retro	Single institution	Other (data mining: empirical decomposition)	U/S	Classification of symptomatic vs. asymptomatic carotid plaque	1,773	76.3%	10-fold cross-validation	Sn 97.3%, Sp 83.2%, Ac 91.4%
Polak 2019[80]	US	Retro	4 clinical trials	Neural network	Duplex velocity measurements	Classification of degree of carotid stenosis (%)	775	NR	NR	Correlation coefficient 0.80
Saba 2019[81]	Japan	Retro	Single institution	CNN	U/S	Classification of degree of carotid stenosis (10% intervals)	407	NR	10-fold cross-validation	AUROC 0.94
Kats 2019[82]	Israel	Retro	Single institution	CNN	Dental panoramic images	Detection of carotid stenosis	65	NR	NR	Sn 75.0%, Sp 80.0%, Ac 83.0%
Verde 2019[83]	Italy	Retro	Public database	ANN	ECG (heart rate variability)	Detection of carotid stenosis	126	70.6%	10-fold cross-validation	AUROC 0.96, Sn 97.7%, Sp 72.9%, Ac 90.5%
Skandha 2020[84]	UK	Retro	Single institution	Multiple (CNN, Naïve Bayes, SVM, k-nearest neighbours, Decision Tree)	U/S	Classification of symptomatic vs. asymptomatic carotid plaque	346	56.6%	10-fold cross-validation	AUROC 0.96, Ac 95.7%
Hsu 2020[85]	US	Retro	Single institution	SVM	U/S	Detection of carotid stenosis	8,211	NR	Leave-one-out cross-validation	Sn 100%, Sp 100%
Yin 2020[86]	China	Retro	Single institution	Random forest	30 demographic/clinical/laboratory variables	Detection of asymptomatic carotid stenosis (screening)	2,841	11.5%	NR	AUROC 0.89
Wu 2020[87]	US	Retro	Single institution	CNN, RNN	U/S report	Detection of carotid stenosis	1,527	26.5%	NR	Sn 84.1%, Sp 98.7%, Ac 95.4%
Zhang 2020[88]	China	Retro	Single institution	Other (Lasso)	Clinical characteristics and MRI data	Classification of symptomatic vs. asymptomatic carotid plaque	162	66.7%	Bootstrapping	AUROC 0.99, Sn 92.6%, Sp 92.9%, Ac 92.7%, NPV 86.7%, PPV 96.2%
Saba 2021[89]	Italy	Retro	Single institution	CNN	U/S	Classification of symptomatic vs. asymptomatic carotid plaque	346	56.6%	10-fold cross-validation	AUROC 0.91, Ac 89.7%, run time < 2s
**Prognosis**										
Aleksic 2008[90]	Germany	Retro	Single institution	ANN	12 peri-operative variables	Prediction of shunt necessity in carotid endarterectomy	850	20.4%	NR	AUROC 0.84, Ac 96.0%, similar to logistic regression: AUROC 0.873, Ac 98.0%,

Author/ Year	Country	Design	Data source	Machine learning model	Input features	Prediction outputs	Sample size	Event rate	Validation method	Outcomes
Okser 2010[91]	Finland	Retro	Population-based study	Bayes classifier (Naïve)	Demographic/clinical/laboratory/genomic variables	Prediction of carotid stenosis progression	813	NR	10-fold cross-validation	AUROC atherosclerosis risk (0.84), progression: (0.76), better compared to cardiovascular risk factors alone: atherosclerosis risk (0.74), progression (0.62)
Kyriacou 2015[92]	UK	Retro	Prospective cohort study	SVM	Clinical and U/S data	Prediction of 3-year stroke in asymptomatic carotid stenosis patients	92	70.7%	NR	AUROC 0.82, Sn 81.%, Sp 73.9%, Ac 76.1%
Hu 2015[93]	US	Retro	Clinical trial	Bayes classifier (Naïve)	115 demographic/clinical/laboratory variables	Prediction of rapid carotid stenosis progression	382	10.2%	3-fold cross-validation	AUROC 0.80, Ac 89.2%
Cheng 2017[94]	Taiwan	Retro	National registry	ANN	13 demographic/clinical variables	Prediction of major adverse cardiovascular events following carotid artery stenting	317	26.5%	NR	AUROC 0.77, Sn 89.4%, Sp 57.4%, Ac 82.5%
Xiao 2018[95]	China	Retro	Single institution	Multiple (SVM, back propagation neural network)	MRI	Prediction of cognitive impairment in patients with carotid stenosis	43	44.2%	NR	AUROC 0.99, Ac 90.0%
Jeon 2018[96]	Korea	Prosp	Single institution	Multiple (ANN, SVM)	Peri-operative demographic/clinical/imaging characteristics	Prediction of hypotension/bradycardia > 1 hour following carotid stent	109	NR	NR	AUROC 0.95, Ac 97.0%, better than logistic regression: AUROC 0.80, Ac 75.8%
Bai 2020[97]	China	Retro	Single institution	Multiple (XGBoost, SVM, decision tree, random forest, ANN)	37 peri-operative variables	Prediction of in-hospital myocardial or cerebral infarction after carotid endarterectomy	443	3.6%	NR	Ac 94.0%
Jamthikar 2020[98]	Japan	Retro	NR	Random forest	13 clinical variables and 25 U/S features	Prediction of stroke risk in carotid stenosis	404	NR	Leave-one-out cross-validation	AUROC 0.99
Tan 2021[99]	China	Retro	Single institution	Ensemble (XGBoost)	100 peri-operative variables from EMR	Prediction of hypertension following carotid endarterectomy	406	13.1%	4-fold cross-validation	AUROC 0.77, Sn 90.0%, Sp 52.0%
**Image segmentation**										
Adame 2004[100]	Netherlands	Retro	Single institution	Fuzzy clustering	MRI	Segmentation of carotid lumen, outer wall, and plaque	10	N/A	NR	Interclass correlations lumen r = 0.92, outer wall r = 0.91, fibrous cap thickness r = 0.71
Molinari 2010[101]	Italy	Retro	Single institution	Fuzzy c-means	U/S	Segmentation of carotid lumen-intima and media-adventitia	135	N/A	NR	Segmentation error 55.6μm for lumen-intima and 34.4μm for media-adventitia, processing time 33.6s
Molinari 2010[102]	Italy	Retro	Single institution	Fuzzy k-means	U/S	Identification of carotid lumen-intima and media-adventitia interfaces	200	N/A	NR	Mean segmentation error 16.2μm, signal to noise radio for lumen-intima (3.6) and media-adventitia (7.6)
Rosati 2011[103]	Italy and Turkey	Retro	2 institutions	ANN	U/S	Identification of carotid lumen, lumen-intima interface, and adventitia	300	N/A	NR	Ac 90.0%
Santhiyakumari 2011[104]	India	Retro	Single institution	ANN	U/S	Segmentation of carotid artery	200	N/A	NR	Ac 96.0%, testing time 0.013s
Molinari 2011[105]	Italy	Retro	2 institutions	Other (greedy approach)	U/S	Segmentation of carotid artery	300	N/A	NR	Segmentation error 0.074mm
Destrempes 2011[106]	Canada	Retro	Single institution	Bayesian model	U/S	Segmentation of carotid plaque, lumen, and adventitia	8,988	N/A	NR	Mean point-to-point distance 0.24mm, Hausdorff distance 1.24mm, segmentation 38s/frame

Author/ Year	Country	Design	Data source	Machine learning model	Input features	Prediction outputs	Sample size	Event rate	Validation method	Outcomes
Milos 2011[107]	Serbia	Retro	NR	Multiple (neural network, k-nearest neighbour)	Virtual models	Segmentation of wall shear stress at carotid bifurcation	4,779	N/A	NR	Root mean square error 0.035 (aneurysm), 0.031 (carotid)
Rocha 2012[108]	Portugal	Retro	Single institution	Fuzzy classifier	U/S	Identification of carotid lumen-intima and media-adventitia interfaces	50	N/A	NR	Coefficient of variation 5.9%-9.0%, mean measurement error 0.14-0.22mm, run time 2.1s
Hassan 2012[109]	Pakistan	Retro	Single institution	Multiple (fuzzy c-means, ensemble clustering, ANN)	U/S	Measurement of carotid intima-media thickness	150	N/A	10-fold cross-validation	Sn 99.2%, Sp 93.9%, Ac 98.4%
Makhijani 2012[110]	US	Retro	NR	Other (hidden Markov tree)	MRI (3D)	Measurement of lumen area, wall area, wall thickness, plaque calcification, and necrotic core area	12	N/A	NR	Mean difference lumen area ($0.17mm^2$), wall area ($-0.27mm^2$), wall thickness (-0.035mm), necrotic core area ($-0.016mm^2$), calcification area ($0.13mm^2$)
Molinari 2012[111]	Italy	Retro	Single institution	Fuzzy K-means	U/S	Measurement of carotid intima-media thickness	885	N/A	NR	Figure of Merit 94.7%, measurement error 0.061mm
Chaudhry 2013[112]	China	Retro	Single institution	SVM	U/S	Segmentation of carotid artery	NR	N/A	Leave-one-out cross-validation	Sn 100%, Sp 97.7%, Ac 98.8%, NPV 100.0%
Hassan 2014[113]	Pakistan	Retro	Single institution	Multiple (fuzzy c-means, probabilistic neural network)	U/S	Measurement of carotid intima-media thickness	300	N/A	NR	AUROC 0.98, Sn 93.4%, Sp 97.6%, Ac 98.4%
Engelen 2014[114]	Netherlands	Retro	Single institution	SVM	MRI/CT	Segmentation of carotid plaque into calcified, fibrous, and lipid-rich necrotic core tissue	13	N/A	Leave-one-out cross-validation	Spearman rank correlation 0.91 (calcification), 0.80 (fibrous), 0.81 (necrotic)
Thornhill 2014[115]	Canada	Retro	Single institution	Multiple (ANN, SVM)	CT	Segmentation of carotid plaque into free-floating thrombus and atherosclerosis	23	N/A	10-fold cross-validation	AUROC 0.85, Sn 87.5%, Sp 71.4%, Ac 76.4%
Rocha 2014[116]	Portugal	Retro	2 institutions	Bayes classifier	U/S	Identification of carotid lumen-intima and media-adventitia interfaces	199	N/A	5-fold cross-validation	Ac 99.5%
Alam 2015[117]	Pakistan	Retro	NR	Fuzzy radial basis function network	U/S	Carotid segmentation and measurement of intima-media thickness	200	N/A	NR	Sn 97.5%, Sp 93.9%, Ac 98.2%
Gao 2015[118]	Netherlands	Retro	NR	Other (supervised multispectral classification)	MRI	Segmentation of carotid lumen, outer wall, and plaque including fibrous tissue, lipid, calcification, ulceration, and hemorrhage	46	N/A	Leave-one-out cross-validation	Spearman's correlation: fibrous tissue (0.81), lipid (0.34), calcification (0.66), loose matrix (0.50)
Huang 2015[119]	China	Retro	Single institution	Multiple (k-nearest neighbours, ANN)	U/S	Segmentation of carotid plaque	218	N/A	NR	Ac 87.5%
Van Engelen 2015[120]	Netherlands	Retro	Prospective cohort study	Other (transfer learning)	MRI	Segmentation of carotid plaque into fibrous tissue, lipid, calcification, and intraplaque hemorrhage	42	N/A	Leave-one-out cross-validation	Sn 96.0%
Kutbay 2016[121]	Turkey	Retro	Single institution	Fuzzy c-means	U/S	Measurement of carotid intima-media thickness	25	N/A	NR	Mean error 0.0014mm
Pazinato 2016[122]	Brazil	Retro	Single institution	SVM	U/S	Segmentation of carotid plaque	12	N/A	NR	Ac 73.2%

Author/ Year	Country	Design	Data source	Machine learning model	Input features	Prediction outputs	Sample size	Event rate	Validation method	Outcomes
Araki 2017[123]	Japan	Retro	Single institution	SVM	U/S	Segmentation of carotid artery	407	N/A	10-fold cross validation	AUROC 0.98, Sn 98.3%, Sp 97.6%, PPV 99.4%, better than manual segmentation: AUROC 0.95, Sn 98.1%, Sp 91.2%, PPV 99.0%
Lekadir 2017[124]	US	Retro	Public database	CNN	U/S	Segmentation of carotid plaque into lipid core, fibrous cap, and calcified tissue	90,000	N/A	NR	Correlation coefficient 0.90
Biswas 2018[125]	Japan	Retro	Single institution	CNN	U/S	Measurement of carotid intima-media thickness	204	N/A	10-fold cross-validation	AUROC 0.88, error 0.12mm, correlation coefficient 0.96
Qian 2018[126]	China	Retro	Public dataset	Multiple (SVM, AdaBoost, random forest)	U/S	Segmentation of carotid vessel wall and plaque	80	N/A	Leave-one-out cross-validation	AUROC 0.90, Sn 80.4%, Sp 96.5%, dice coefficient 81.0%
Hassan 2019[127]	Pakistan	Retro	Single institution	Multiple (spatial fuzzy c-means, Gaussian mixture model, SVM)	U/S	Measurement of carotid intima-media thickness	250	N/A	NR	Sn 99.3%, Sp 98.0%, Ac 98.8%
Savas 2019[128]	Turkey	Retro	Single institution	CNN	U/S	Measurement of carotid intima-media thickness	501	N/A	NR	Sn 89.0%, Sp 88.0%, Ac 89.1%
Wu 2019[129]	China	Retro	2 datasets: 13 institutions and 21 institutions	CNN (Deep U-shape)	MRI	Segmentation of carotid lumen, outer wall, vessel wall, and atherosclerosis	1057 (development), 425 (external validation)	N/A	10-fold cross-validation	Development (AUROC 0.95, Ac 89.2%, dice coefficient 0.86-0.96, external validation (AUROC 0.92, Ac 86.8%, dice coefficient 0.84-0.95
Zhang 2019[130]	China	Retro	Single institution	Multiple (Naïve Bayes, SVM, random forest, gradient boosting decision tree, ANN)	MRI	Segmentation of carotid plaque into lipid rich/necrotic core, intraplaque hemorrhage, calcification, and fibrous tissue	68	N/A	10-fold cross-validation	Ac 88.0%, correlation coefficient 0.82
Roy-Cardinal 2019[131]	Canada	Retro	Single institution	Random forest	U/S	Segmentation of carotid plaque into lipid, calcification, and fibrous cap	66	39.4%	Bootstrapping	AUROC (lipid: 0.90, calcification: 0.95)
Zhou 2019[132]	China	Retro	Single institution	CNN (dynamic U-Net)	U/S (3D)	Carotid segmentation and identification of media-adventitia and lumen-intima boundaries	38	N/A	3-fold cross-validation	Dice coefficient 96.5%
Tsakanikas 2020[133]	Greece	Retro	Single institution	CNN (U-net)	MRI	3D reconstruction of carotid artery	30	N/A	NR	Ac (lumen area: 99.1%, lumen perimeter: 92.6%)
Mi 2020[134]	China	Retro	NR	CNN	U/S	Identification of carotid intima-media interface	23	N/A	NR	Ac 86.0%
Zhou 2020[135]	China	Retro	Single institution	CNN	U/S	Identification of carotid media-adventitia and lumen-intima boundaries	1,007	N/A	NR	Dice coefficient 93.2% (common carotid artery), 91.9% (bifurcation), run time < 1s
Vila 2020[136]	Spain	Retro	2 datasets: 1 national registry and 1 single institution	CNN (DenseNets)	U/S	Measurement of carotid intima-media thickness	2,379	N/A	NR	Common carotid artery (Sn 80.0%, Sp 96.6% Ac 96.5%), carotid bulb (Sn 78.3%, Sp 75.0%, Ac 78.1%), correlation coefficient 0.81
Biswas 2020[137]	Japan	Retro	Single institution	CNN	U/S	Measurement of carotid intima-media thickness and total plaque area	250	N/A	10-fold cross-validation	AUROC 0.87, carotid intima thickness error 0.094 mm, total plaque area error 2.79 mm2, correlation coefficient

Author/ Year	Country	Design	Data source	Machine learning model	Input features	Prediction outputs	Sample size	Event rate	Validation method	Outcomes
										(carotid intima media thickness 0.99, plaque area 0.89)
Meshram 2020[138]	US	Retro	Single institution	CNN (dilated U-Net)	U/S	Segmentation of carotid artery	101	N/A	NR	Dice coefficient 0.83
He 2020[139]	China	Retro	Single institution	Random forest	Optical coherence tomography of ex vivo carotid plaque tissue	Segmentation of carotid plaque components including fibrous, calcified, and lipid tissue	31	N/A	10-fold cross-validation	Ac (fibrous: 80.0%, calcified: 62.0%, lipid: 83.1%)
Zhou 2021[140]	China and Canada	Retro	2 datasets from 2 single institutions	CNN (U-Net)	U/S	Carotid segmentation and measurement of total plaque area	144 (development), 497 (external validation)	N/A	3-fold cross-validation	Dice coefficient 83.3-85.7%, Pearson's correlation r = 0.985-0.988, intra-class correlation ICC = 0.996
Zhou 2021[141]	China	Retro	Public database	CNN (3D deep learning U-Net)	CT	Segmentation of carotid artery	56	N/A	Leave-one-out cross-validation	Dice coefficient 82.3%
Zhao 2021[142]	China	Retro	Single institution	Other (multitask regression network)	U/S	Segmentation of carotid artery	101	N/A	5-fold cross-validation	Dice coefficient 84.0%
**Diabetic foot ulcer**										
**Diagnosis**										
Acharya 2008[143]	Singapore	Retro	Single institution	Neural network (back propagation)	Foot scan (F-scan pressure measurement system)	Detection of diabetic neuropathy	84	33.3%	NR	AUROC 0.86, Sn 100%, Sp 92.8%, PPV 87.5%
Acharya 2012[144]	Singapore	Retro	Single institution	Fuzzy classifier	Planter pressure images	Detection of diabetic neuropathy	91	20.9%	6-fold cross-validation	Sn 100%, Sp 83.3%, Ac 93.7%
Li 2017[145]	US	Retro	Single institution	CNN	Foot scan	Detection of diabetic plantar pressure	10	NR	NR	Ac 80.0%, root mean square error 70%
Huang 2018[146]	China	Retro	NR	Neural network (radial basis function)	Hemoglobin concentration and oxygen saturation on foot	Detection of DFU	45	NR	NR	NR
Han 2020[147]	China	Retro	Single institution	CNN (Faster R-CNN)	DFU pictures	Classification of DFU severity (Wagner grades 1-6)	2,688	NR	5-fold cross-validation	Precision 91.4%
Goyal 2020[148]	UK	Retro	Single institution	Ensemble CNN (Inception-V3, ResNet50, InceptionResNetV2)	DFU pictures	Detection of DFU infection or ischemia	1,459	85.6%	5-fold cross-validation	Ischemia (AUROC 0.90, Sn 88.6%, Sp 92.1%, Ac 90.3%), infection (AUROC 0.73, Sn 70.9%, Sp 74.4%, Ac 72.7%)
**Prognosis**										
Singh 2013[149]	India	Retro	Single institution	ANN	5 single nucleotide polymorphisms	Prediction of DFU development	255	49.0%	NR	Ac 83.0%, better than linear regression 76%
Lopez-de-Andres 2016[150]	Spain	Retro	National registry	ANN	4 demographic/clinical variables	Prediction of in-hospital mortality following major lower extremity amputation	40,857	NR	Quasi-Newton method	Sn 76.3%, Sp 96.0%, Ac 86.1%
Nguyen 2020[151]	US	Retro	Single institution and public database	Mutiple (XGBoost, multilayer perceptron ANN, decision tree, SVM)	Wound pictures	Prediction of need for referral to wound care specialist	205	NR	10-fold cross-validation	Ac 81.0%

Author/ Year	Country	Design	Data source	Machine learning model	Input features	Prediction outputs	Sample size	Event rate	Validation method	Outcomes
Lin 2020[152]	China	Retro	Single institution	Neural network (back propagation)	33 clinical/laboratory variables	Prediction of 3-year mortality in patients with DFU	200	NR	NR	AUROC 0.71, Sn 73.9%, Sp 70.6%, better compared to cox regression: AUROC 0.64, Sn 26.3%, Sp 89.5%
Kim 2020[153]	US	Retro	Single institution	Multiple (random forest, SVM)	48 clinical variables from EMR and 2,048 deep-learning features from DFU images	Prediction of DFU healing	208	NR	3-fold cross-validation	AUROC 0.73, Ac 81.1%
Yang 2021[154]	US	Retro	National registry	Multiple (LASSO, gradient boosting, random forest)	41 demographic/clinical variables	Prediction of lower extremity amputation in patients with diabetes prescribed canagliflozin	13,904	0.6%	NR	AUROC 0.81
Ravaut 2021[155]	Canada	Retro	Registry (provincial)	Decision tree (gradient boosted)	700 demographic/clinical	Prediction of complications in patients with diabetes including amputation	1,567,636	NR	Single fixed validation set	AUROC 0.78, appropriate calibration curves
**Image segmentation**										
Yavuz 2009[156]	US	Retro	NR	Multiple (neural network (feed-forward) and fuzzy logic)	Plantar pressure and shear stress during ambulation	Segmentation of foot plantar pressure	73	69.9%	NR	Root mean square error 0.27 - 0.40
Mukherje e 2014[157]	India	Retro	Public database (Medetec)	Multiple (SVM, Bayesian classification)	Wound pictures	Segmentation of DFU into granulation, necrotic, or slough tissue	767	N/A	NR	Ac (granulation: 86.9%, slough: 90.5%, necrotic: 75.5%)
Liu 2015[158]	Netherla nds	Retro	Single institution	Other (k-means clustering)	DFU thermogram pictures	Segmentation of DFU by temperature	76	N/A	NR	Sn 97.8%, Sp 98.4%
Wang 2017[159]	US	Retro	Single institution	SVM	DFU pictures (smartphone)	Localization and segmentation of DFU	100	N/A	k-fold cross-validation	Sn 73.3%, Sp 94.6%
Babu 2018[160]	India	Retro	Public database	Fuzzy C-means and K-means	Wound pictures	Localization and segmentation of DFU	5	N/A	NR	Ac 96.8%, mean square error 0.19
Cui 2019[161]	US	Retro	Single institution	CNN	Wound pictures	Localization and segmentation of DFU	445	N/A	4-fold cross-validation	Sn 93.7%, Sp 96.0%, Ac 93.9%, dice coefficient 0.82
Ohura 2019[162]	Japan	Retro	Single institution	CNN (SegNet, LinkNet, U-Net)	Wound pictures	Localization of DFU	440	N/A	NR	AUROC 1.0, Sn 99.3%, Sp 94.3%
Goyal 2019[163]	UK	Retro	Multiple institutions	CNN (Faster R-CNN)	DFU pictures	Localization of DFU	1,775	N/A	5-fold cross-validation	Precision 91.8%, speed 48ms
Wang 2020[164]	China	Retro	Single institution with validation on public dataset	CNN (Deep, MobileNetV2)	Wound pictures	Localization and segmentation of DFU	1109	N/A	NR	Dice coefficient/precision/recall: development (90.5%/94.3%/91.3%), external validation (94.1%/98.4%/94.3%)
Cruz-Vega 2020[165]	Mexico	Retro	Public database	Multiple (SVM, multilayer perceptron neural network, CNN)	DFU thermogram pictures	Segmentation of DFU by temperature	110	N/A	10-fold cross-validation	AUROC 0.95, Sn 95.3%, Sp 93.8%, Ac 94.4%
Zoppo 2020[166]	Italy	Retro	Clinical trial	Other (machine learning)	Wound pictures	Segmentation of DFU into granular, slough, or necrotic tissue	150	N/A	NR	Ac 97.0%
**Peripheral artery disease**										
**Diagnosis**										
Gindi 1991[167]	US	Retro	NR	Other (k-nearest neighbours)	Fluoroscopy	Detection of lower extremity atherosclerosis	154	NR	NR	Ac 96.0%

Author/ Year	Country	Design	Data source	Machine learning model	Input features	Prediction outputs	Sample size	Event rate	Validation method	Outcomes
Karamchandani 2005[168]	India	Retro	NR	ANN	Impedance cardio-vasography	Detection of PAD	100	70.0%	NR	NR
Huang 2011[169]	China	Prosp	Single institution	Other (Body area sensor network)	Gait information	Classification of gait pattern	912	NR	NR	Ac 89.1%, mean square error 0.13
Watanabe 2013[170]	Japan	Retro	NR	SVM	Video capture of gait	Classification of ischemic vs. neurogenic claudication	59	22.0%	Leave-one-out cross-validation	Ac 79.7%
Watanabe 2014[171]	Japan	Retro	NR	Multiple (SVM, decision tree)	Walking motion data	Classification of ischemic vs. neurogenic claudication	46	21.7%	Leave-one-out cross-validation	Ac 83.0%
Li 2014[172]	Taiwan	Retro	Single institution	SVM	Photoplethysmography	Classification of PAD severity (normal/low/high degree)	33	51.5%	NR	Ac 80.0%
LeMoyne 2015[173]	US	Retro	Single institution	SVM	Force plate recordings during stance phase of gait	Detection of prosthesis type (manual vs. powered)	1	NR	Leave-one-out cross-validation	Ac 100%
Afzal 2016[174]	US	Retro	Single institution	Other (Apache Unstructured Information Management Architecture)	Clinical notes from EMR	Detection of PAD	117	23.1%	NR	Sn 96.0%, Sp 98.0%, PPV 92.0%, NPV 99.0% Ac 100%
Shawen 2017[175]	US	Retro	Single institution	Multiple (random forest, SVM, XGBoost)	Accelerometer and gyroscope sensor of mobile phone	Detection of falls	16 (development), 3 (external validation)	NR	Leave-one-out cross-validation	Development (AUROC 1.0, Sn 98.3%, Sp 99.1%), external validation (AUROC 0.99, Sn 97.0%, Sp 95.0%)
McCarthy 2018[176]	US	Retro	Prospective cohort study	Other (Lasso)	50 clinical variables and 109 genomic biomarkers	Detection of PAD	354	37.3%	Monte Carlo cross-validation	AUROC 0.85, Sn 65.0%, Sp 88.0%, PPV 76.0%, NPV 81.0%
Jana 2019[177]	India	Retro	NR	Multiple (random forest, SVM)	Doppler U/S	Classification of degree of lower extremity artery stenosis (normal/stenosis/occlusion)	334	NR	10-fold cross validation	Ac 97.9%, PPV 98.0%
Kim 2020[178]	China	Retro	Single institution	CNN	Arterial pulse waveforms	Detection of PAD	253,125	NR	10-fold cross-validation	AUROC 0.99, Sn 85.0%, Sp 99.0%, Ac 91.0%
Weissler 2020[179]	US	Retro	Single institution	Neural network (multilayer perceptron)	Clinical notes from EMR	Detection of PAD	6,861	54.6%	10-fold cross validation	AUROC 0.89, Sp 62.0%, precision 74%, better compared to logistic regression AUROC/precision/Sp (0.82/65%/41%)
Qutrio Baloch 2020[180]	US	Retro	NR	Multiple (random forest, neural network, generalized linear model)	Demographic/clinical variables	Detection of critical limb ischemia	703	19.8%	NR	AUROC 0.69
Dai 2021[181]	China	Retro	Single institution	CNN (parallel efficient network, p-EffNet)	CT	Classification of degree of lower extremity arterial stenosis	265	NR	NR	Above knee artery (AUROC 0.99, Sn 90.2%, Sp 97.7%, Ac 91.5%), below knee artery (AUROC 0.98, Sn 91.3%, Sp 95.2%, Ac 90.9%)
Stolyarov 2021[182]	US	Prosp	Single institution	Decision tree	Gait information from prosthesis sensors	Detection of terrain type (ramp, staircase, flat ground) for prosthesis	1,594	NR	10-fold cross-validation	Prediction error 2.8%

Author/ Year	Country	Design	Data source	Machine learning model	Input features	Prediction outputs	Sample size	Event rate	Validation method	Outcomes
Ross 2016[183]	US	Retro	Prospective cohort study	Multiple (elastic net, penalized regression mode, random forest)	130 demographic/clinical/imaging/genomic variables	Detection of PAD and prediction of mortality risk	1,047	12.3%	10-fold cross-validation	PAD identification (AUROC 0.87), mortality prediction (AUROC 0.76), Hosmer-Lemeshow test (P = 0.7), better than logistic regression: PAD identification (AUROC 0.76), mortality prediction (AUROC 0.65)
**Prognosis**										
Yurtkuran 2013[184]	Turkey	Retro	Single institution	Neural network (radial basis function)	16 demographic/clinical variables	Prediction of need for surgical treatment in patients with PAD	186	52.2%	10-fold cross-validation	AUROC 0.95, Sn 95.3%, Sp 94.8%, Ac 95.0%, PPV 94.2%, NPV 95.8%
Knezevic 2016[185]	Serbia	Retro	Single institution	SVM	11 demographic/clinical variables	Prediction of ambulation following lower extremity amputation	263	66.2%	NR	Ac 88.9%
Wurdeman 2019[186]	US	Retro	Multiple institutions	Decision tree (Classification and regression tree)	6 clinical variables	Prediction of ambulation in patients with lower limb prosthesis	2,770	NR	NR	Ac 81.6%
Ross 2019[187]	US	Retro	2 institutions	Multiple (penalized linear regression, random forest)	957 demographic/clinical/laboratory variables and unstructured clinical notes from EMR data	Prediction of major adverse cardiac and cerebrovascular events in PAD patients	7,686	16.9%	5-fold cross-validation	AUROC 0.81, Sn 50.0%, Sp 96.0%, PPV 80.0%, NPV 90.0%, calibration Brier score 0.10
Keles 2020[188]	US	Retro	Single institution	Neural network (multilayer perceptron)	Surface electromyogram	Prediction of ankle prosthesis position and momentum	40	NR	NR	Pearson's correlation coefficient 0.99, root mean square error 0.17
Berger 2020[189]	US	Retro	National registry	Bayesian model	Demographic/clinical variables	Prediction of 1-year all-cause hospitalization and total annual healthcare cost in PAD patients	3,189	60.0%	5-fold cross-validation	AUROC 0.63
Chang 2020[190]	US	Retro	2 datasets: national registry and single institution	Neural network (multilayer perceptron)	Peri-operative variables from EMR	Prediction of surgical site infection after vascular surgery	72,435 (development), 370 (external validation)	12.4%	NR	AUROC 0.61
Perkins 2020[191]	US	Retro	2 national registries	Bayes classifier (Naïve)	10 clinical variables	Prediction of failed revascularization requiring amputation	508 (development), 51 (external validation)	12.9%	10-fold cross-validation	Development (AUROC 0.95, calibration slope 1.96), external validation (AUROC 0.97, calibration slope 1.72), AUROC better than mangled extremity severity score 0.74
Bolourani 2021[192]	US	Retro	National registry	Ensemble (XGBoost, random forest, logistic regression)	Peri-operative variables	Prediction of 30-day amputation following lower extremity trauma	1,098	18.8%	4-fold cross-validation	Sn 47.0%, Sp 98.0%, Ac 88.0%
**Image segmentation**										
Kinner 2011[193]	Germany	Retro	Single institution	Other (k-space segmentation)	MRI	Segmentation of peripheral arteries	20	N/A	NR	Mean score 3.0
Mandelias 2013[194]	Greece	Retro	Single institution	Fuzzy c-means	Intravascular optical computed tomography	Identification of lower extremity artery lumen and stent struts	4	N/A	NR	Sn 66.0%, Sp 94.1%, Ac 93.8%, overlap coefficient 0.92, mean difference 0.089mm, processing time 4.5s/frame
Zhang 2020[195]	US	Retro	Single institution	ANN	MRI	Segmentation of calf muscle hyperemia	48	56.3%	NR	Correlation coefficient 0.95, run time 1s, run time better than manual model fitting of 80 min

Author/ Year	Country	Design	Data source	Machine learning model	Input features	Prediction outputs	Sample size	Event rate	Validation method	Outcomes
Hippe 2020[196]	US	Retro	Clinical trial	CNN	MRI	Segmentation of popliteal artery and measurement of wall thickness and lumen area	4,796	N/A	NR	Coefficient of variation for mean wall thickness 4.7%, mean lumen area 4.6%
**Renal artery stenosis**										
**Diagnosis**										
Jaulent 1997[197]	France	Retro	Multiple institutions	Fuzzy clustering	Completion angiogram	Detection of renal artery angioplasty success	50	NR	NR	NR
Nielsen 2005[198]	Sweden	Retro	Single institution	ANN	Captopril renography	Detection of renal artery stenosis	250	21.2%	8-fold cross validation	AUROC 0.93, Sn 91.0%, Sp 90.0%
**Prognosis**										
Chen 2019[199]	US	Retro	Clinical trial	Multiple (SVM, decision tree, feed-forward neural network, random forest)	Demographic/clinical/laboratory characteristics	Prediction of major cardiovascular or renal events in patients with renal artery stenosis	573	NR	NR	AUROC 0.68, Sn 30.0%, Sp 96.0%, Ac 74.0%
**Image segmentation**										
Lalande 1999[200]	France	Retro	NR	Fuzzy logic	Fluoroscopy	Segmentation of renal artery and measurement of degree of stenosis	29	N/A	NR	NR
**Venous disease**										
**Diagnosis**										
Wang 2020[201]	China	Retro	Single institution	Multiple (random forest, decision tree, k-nearest neighbour, SVM, Naïve Bayes, XGBoost)	22 demographic/clinical variables from EMR	Detection of venous thromboembolism	376 (development), 1579 (external validation)	50.0%	10-fold cross-validation	Development (AUROC 0.86, Sn 73.3%, Sp 80.0%, PPV 0.79), external validation (AUROC 0.78, Sn 59.5%, Sp 86.5%, PPV 0.11), better than traditional Padua prediction score AUROC 0.74, Sp 0.51
**Prognosis**										
Fukaya 2019[202]	UK	Retro	Public database (UK Biobank)	Other (gradient boosting machine model)	2,715 demographic/clinical variables and 10,972,371 genomic markers	Prediction of varicose vein development	493,519	1.9%	NR	AUROC 0.70
Taylor 2002[203]	UK	Retro	Single institution	ANN	Clinical variables and ulcer characteristics	Prediction of healing time for venous ulcers	325	NR	NR	Ac 68.0%
Franciscis 2016[204]	Italy	Retro	Multiple institutions	Fuzzy logic	Demographic/clinical variables and U/S characteristics	Prediction of venous ulcer development	77	51.9%	NR	NR
**Other vascular conditions**										
**Diagnosis**										
Zhao 2018[205]	Canada	Retro	NR	Multiple (Random forest, SVM, k-nearest neighbours)	3D vertebral artery aneurysm images	Prediction of vertebral artery aneurysm rupture	37	32.4%	Leave-one-out cross-validation	AUROC 0.85, Sn 75.0%, Sp 84.0%, Ac 81.4%
Karhade 2020[206]	US	Retro	5 institutions	Multiple (gradient boosting, random forest, SVM, neural network, penalized logistic regression, XGBoost)	Peri-operative variables and operative note	Detection and prediction of vascular injury in anterior lumbar spine surgery	1035	7.2%	NR	Detection (AUROC 0.92, Sn 86.0%, Sp 93.0%, NPV 0.99, PPV 0.51, calibration slope 0.61), prediction (AUROC 0.73, Sn 81.0%, Sp 57.0%, NPV 0.97, PPV 0.15 calibration slope

Author/ Year	Country	Design	Data source	Machine learning model	Input features	Prediction outputs	Sample size	Event rate	Validation method	Outcomes
										1.43), AUROC for detection better than administrative codes of 0.64
Yeih 2014[207]	Taiwan	Retro	NR	SVM	Electronic stethoscopes signals	Detection of arteriovenous fistula stenosis > 50%	50	NR	NR	Sn 86.2%, Sp 95.2%, Ac 90.0%, PPV 96.2%, NPV 83.3%
Kistenev 2019[208]	Russia	Retro	Single institution	SVM	Tissue analysis	Detection of lymphedema	78	46.2%	Leave-one-out cross-validation	Sn 79.0%, Sp 77.0%, Ac 96.0%
**Prognosis**										
Lapuerta 1998[209]	US	Retro	2 datasets: development (2 institutions) external validation (3 institutions)	Neural network (back propagation)	Clinical variables, ECG findings, dipyridamole thallium results	Prediction of in-hospital myocardial infarction and cardiac death in vascular surgery patients	567 (development), 514 (external validation)	8.0%	m-items-out	Development (AUROC 0.76, Hosmer-Lemeshow calibration statistic 18.6), external validation (AUROC 0.68, calibration statistic 15.4), better compared to logistic regression: development (AUROC 0.76, calibration statistic 45.0), external validation (AUROC 0.68), calibration statistic 55.9)
Zhao 2019[210]	China	Prosp	NR	CNN	Simulated endovascular cases	Prediction of endovascular catheter movement	200	NR	NR	Ac 100%
Amato 2020[211]	US	Retro	Registry (statewide)	Multiple (random forest, discriminant analysis, gradient boosting)	Peri-operative variables	Prediction of 90-day hospital readmission after major vascular surgery	246,405	30.3%	NR	AUROC 0.68, PPV 0.74, NPV 0.55
**Image segmentation**										
Zhou 2020[212]	China	Retro	Single institution	Neural network (pyramid attention recurrent network)	Fluoroscopy	Real-time guidewire segmentation and tracking	30	N/A	4-fold cross-validation	F score 0.94

* Retro (retrospective), Prosp (prospective), SVM (support vector machine), CNN (convolutional neural network), ANN (artificial neural network), CT (computed tomography), EMR (electronic medical record), MRI (magnetic resonance imaging), U/S (ultrasound), DFU (diabetic foot ulcer), AAA (abdominal aortic aneurysm), Ac (accuracy), Sn (sensitivity), Sp (specificity), PPV (positive predictive value), NPV (negative predictive value), AUROC (area under receiver operating characteristic curve), NR (not reported), N/A (not applicable)

Supplementary Table 2. Search strategies for A) MEDLINE, B) Embase, and C) Cochrane CENTRAL

A) All Ovid MEDLINE <January 1, 1946 – March 1, 2021>

1	Aortic Aneurysm, Abdominal/ (19826)
2	Aortic Aneurysm, Thoracic/ (12909)
3	Iliac Aneurysm/ (1467)
4	exp Carotid Artery Diseases/ (48585)
5	Diabetic Foot/ (9233)
6	exp Lymphedema/ (12471)
7	Mesenteric Ischemia/ (1140)
8	Peripheral Arterial Disease/ (8459)
9	exp Varicose Veins/ (18210)
10	exp Venous Insufficiency/ (7735)
11	venous disease*.tw,kf. (2860)
12	((aort* or artery) adj3 aneurysm*).tw,kf. (56861)
13	iliac aneurysm*.tw,kf. (825)
14	popliteal aneurysm*.tw,kf. (599)
15	subclavian aneurysm*.tw,kf. (125)
16	brachial aneurysm*.tw,kf. (23)
17	peripheral artery aneurysm*.tw,kf. (65)
18	aort* rupture*.tw,kf. (2495)
19	Loeys-Dietz.tw,kf. (548)
20	((aort* or artery or blood vessel*) adj5 dissection*).tw,kf. (25385)
21	endoleak*.tw,kf. (5292)
22	perigraft leak*.tw,kf. (58)
23	(carotid adj3 (disease* or disorder* or atherosclero* or arteriopath* or fistula* or injur* or trauma* or thrombos?s or stenos?s or narrowing* or plaque* or ulcer*)).tw,kf. (32058)
24	(diabetic foot or diabetic feet).tw,kf. (9166)
25	foot ulcer*.tw,kf. (6639)
26	lymph?edema*.tw,kf. (10834)
27	(mesenteric adj3 (isch?emia* or insufficienc* or thrombos?s or embolus)).tw,kf. (4893)
28	peripheral arter* disease*.tw,kf. (15623)
29	(varicos* or varix or varices or microvaricos*).tw,kf. (32120)
30	(phlebectasi* or vein ectasi*).tw,kf. (325)
31	((venous or stasis) adj3 ulcer*).tw,kf. (5065)
32	venous insufficienc*.tw,kf. (5236)
33	1 or 2 or 3 or 4 or 5 or 6 or 7 or 8 or 9 or 10 or 11 or 12 or 13 or 14 or 15 or 16 or 17 or 18 or 19 or 20 or 21 or 22 or 23 or 24 or 25 or 26 or 27 or 28 or 29 or 30 or 31 or 32 (241752)
34	Endarterectomy, Carotid/ (8994)
35	Axillofemoral Bypass Grafting/ (19)
36	Amputation/ (21454)
37	((vascular or blood vessel or aort* or endovascular or vein) adj3 surg*).tw,kf. (47086)
38	amputation*.tw,kf. (40860)
39	limb salvage*.tw,kf. (7242)
40	aneurysmectom*.tw,kf. (1614)
41	lower extremity bypass*.tw,kf. (439)
42	infra?inguinal bypass*.tw,kf. (773)

43	(popliteal bypass* or femoro?popliteal bypass*).tw,kf. (1253)
44	aortofemoral bypass*.tw,kf. (383)
45	aortobifemoral bypass*.tw,kf. (479)
46	axillo?femoral bypass*.tw,kf. (309)
47	thoracofemoral bypass*.tw,kf. (8)
48	femoro?femoral bypass*.tw,kf. (307)
49	(femorotibial bypass* or tibial bypass*).tw,kf. (321)
50	pedal bypass*.tw,kf. (104)
51	(endarter?ectom* or thromboendarter?ectom*).tw,kf. (16332)
52	vein stripping*.tw,kf. (286)
53	endovenous.tw,kf. (1989)
54	embolectom*.tw,kf. (3320)
55	(angioplast* or endoluminal repair* or transluminal arterial dilation* or balloon dilation*).tw,kf. (48951)
56	atherectom*.tw,kf. (3259)
57	angioscop*.tw,kf. (1258)
58	(mechanical thrombolys?s or mechanical clot disruption*).tw,kf. (286)
59	carotid stent*.tw,kf. (2101)
60	34 or 35 or 36 or 37 or 38 or 39 or 40 or 41 or 42 or 43 or 44 or 45 or 46 or 47 or 48 or 49 or 50 or 51 or 52 or 53 or 54 or 55 or 56 or 57 or 58 or 59 (167726)
61	33 or 60 (368437)
62	exp Artificial Intelligence/ (107813)
63	((artificial or computational or machine or ambient) adj3 intelligence).tw,kf. (12691)
64	AI.tw,kf. (29174)
65	((computer or automated or machine) adj2 (reasoning or inference*)).tw,kf. (278)
66	computer vision system*.tw,kf. (246)
67	(multi?criteria decision* or multiple criteria decision*).tw,kf. (596)
68	machine learning.tw,kf. (40037)
69	transfer learning.tw,kf. (1706)
70	deep learning.tw,kf. (15604)
71	hierarchical learning.tw,kf. (65)
72	semi?supervised learning.tw,kf. (139)
73	support vector*.tw,kf. (18613)
74	(heuristic* or hyperheuristic* or metaheuristic*).tw,kf. (12904)
75	expert system*.tw,kf. (3096)
76	fuzzy logic.tw,kf. (2042)
77	knowledge?base*.tw,kf. (944)
78	natural language processing*.tw,kf. (4084)
79	(neural network* or perceptron*).tw,kf. (58586)
80	connectionist model*.tw,kf. (496)
81	(robotics or remote operation* or telerobotic*).tw,kf. (9068)
82	segmentation.tw,kf. (39020)
83	big data.tw,kf. (8382)
84	(algorithm* adj4 computer*).tw,kf. (4848)
85	62 or 63 or 64 or 65 or 66 or 67 or 68 or 69 or 70 or 71 or 72 or 73 or 74 or 75 or 76 or 77 or 78 or 79 or 80 or 81 or 82 or 83 or 84 (265870)
86	61 and 85 (1760)
87	exp animals/ not humans.sh. (4793649)
88	86 not 87 (1677)
89	limit 88 to (comment or editorial or letter) (32)

90	88 not 89 (1645)

B) Embase Classic and Embase <January 1, 1947 – March 1, 2021>

1	exp abdominal aortic aneurysm/ (6816)
2	exp thoracic aorta aneurysm/ (9107)
3	iliac artery aneurysm/ (1345)
4	popliteal artery aneurysm/ (667)
5	exp carotid artery disease/ (74151)
6	diabetic foot/ (16730)
7	exp lymphedema/ (24119)
8	mesenteric ischemia/ (2799)
9	exp peripheral occlusive artery disease/ (187583)
10	exp varicosis/ (62578)
11	exp vein insufficiency/ (11403)
12	venous disease*.tw,kw. (4500)
13	((aort* or artery) adj3 aneurysm*).tw,kw. (75225)
14	iliac aneurysm*.tw,kw. (1194)
15	popliteal aneurysm*.tw,kw. (800)
16	subclavian aneurysm*.tw,kw. (162)
17	brachial aneurysm*.tw,kw. (23)
18	peripheral artery aneurysm*.tw,kw. (103)
19	aort* rupture*.tw,kw. (3403)
20	Loeys-Dietz.tw,kw. (842)
21	((aort* or artery or blood vessel*) adj5 dissection*).tw,kw. (36037)
22	endoleak*.tw,kw. (7778)
23	perigraft leak*.tw,kw. (73)
24	(carotid adj3 (disease* or disorder* or atherosclero* or arteriopath* or fistula* or injur* or trauma* or thrombos?s or stenos?s or narrowing* or plaque* or ulcer*)).tw,kw. (49095)
25	(diabetic foot or diabetic feet).tw,kw. (13650)
26	foot ulcer*.tw,kw. (9423)
27	lymph?edema*.tw,kw. (15992)
28	(mesenteric adj3 (isch?emia* or insufficienc* or thrombos?s or embolus)).tw,kw. (7343)
29	peripheral arter* disease*.tw,kw. (25540)
30	(varicos* or varix or varices or microvaricos*).tw,kw. (48431)
31	(phlebectasi* or vein ectasi*).tw,kw. (486)
32	((venous or stasis) adj3 ulcer*).tw,kw. (7270)
33	venous insufficienc*.tw,kw. (8476)
34	1 or 2 or 3 or 4 or 5 or 6 or 7 or 8 or 9 or 10 or 11 or 12 or 13 or 14 or 15 or 16 or 17 or 18 or 19 or 20 or 21 or 22 or 23 or 24 or 25 or 26 or 27 or 28 or 29 or 30 or 31 or 32 or 33 (480399)
35	carotid endarterectomy/ (19060)
36	axillofemoral artery anastomosis/ (714)
37	amputation/ (30284)
38	((vascular or blood vessel or aort* or endovascular or vein) adj3 surg*).tw,kw. (72212)
39	amputation*.tw,kw. (58040)
40	limb salvage*.tw,kw. (9474)
41	aneurysmectom*.tw,kw. (2064)
42	lower extremity bypass*.tw,kw. (668)
43	infra?inguinal bypass*.tw,kw. (992)

44	(popliteal bypass* or femoro?popliteal bypass*).tw,kw. (1691)
45	aortofemoral bypass*.tw,kw. (482)
46	aortobifemoral bypass*.tw,kw. (609)
47	axillo?femoral bypass*.tw,kw. (373)
48	thoracofemoral bypass*.tw,kw. (12)
49	femoro?femoral bypass*.tw,kw. (386)
50	(femorotibial bypass* or tibial bypass*).tw,kw. (377)
51	pedal bypass*.tw,kw. (134)
52	(endarter?ectom* or thromboendarter?ectom*).tw,kw. (24070)
53	vein stripping*.tw,kw. (419)
54	endovenous.tw,kw. (3580)
55	embolectom*.tw,kw. (5176)
56	(angioplast* or endoluminal repair* or transluminal arterial dilation* or balloon dilation*).tw,kw. (74756)
57	atherectom*.tw,kw. (4939)
58	angioscop*.tw,kw. (1708)
59	(mechanical thrombolys?s or mechanical clot disruption*).tw,kw. (476)
60	carotid stent*.tw,kw. (3594)
61	35 or 36 or 37 or 38 or 39 or 40 or 41 or 42 or 43 or 44 or 45 or 46 or 47 or 48 or 49 or 50 or 51 or 52 or 53 or 54 or 55 or 56 or 57 or 58 or 59 or 60 (247759)
62	34 or 61 (650442)
63	exp artificial intelligence/ (45948)
64	((artificial or computational or machine or ambient) adj3 intelligence).tw,kw. (16298)
65	AI.tw,kw. (41545)
66	((computer or automated or machine) adj2 (reasoning or inference*)).tw,kw. (303)
67	computer vision system*.tw,kw. (245)
68	(multi?criteria decision* or multiple criteria decision*).tw,kw. (811)
69	machine learning.tw,kw. (49098)
70	transfer learning.tw,kw. (1922)
71	deep learning.tw,kw. (18811)
72	hierarchical learning.tw,kw. (77)
73	semi?supervised learning.tw,kw. (128)
74	support vector*.tw,kw. (23346)
75	(heuristic* or hyperheuristic* or metaheuristic*).tw,kw. (14216)
76	expert system*.tw,kw. (4463)
77	fuzzy logic.tw,kw. (2750)
78	knowledge?base*.tw,kw. (1379)
79	natural language processing*.tw,kw. (5130)
80	(neural network* or perceptron*).tw,kw. (73309)
81	connectionist model*.tw,kw. (573)
82	(robotics or remote operation* or telerobotic*).tw,kw. (12139)
83	segmentation.tw,kw. (54284)
84	big data.tw,kw. (10352)
85	(algorithm* adj4 computer*).tw,kw. (6408)
86	63 or 64 or 65 or 66 or 67 or 68 or 69 or 70 or 71 or 72 or 73 or 74 or 75 or 76 or 77 or 78 or 79 or 80 or 81 or 82 or 83 or 84 or 85 (299449)
87	62 and 86 (2754)
88	(exp animal/ or animal experiment/ or nonhuman/) not (exp human/ or human experiment/) (7473946)
89	87 not 88 (2566)
90	limit 89 to (editorial or letter) (36)

91	89 not 90 (2530)
92	limit 91 to embase (1463)

C) Evidence-Based Medicine Reviews - Cochrane Central Register of Controlled Trials <January 1, 1947 – March 1, 2021>

1	Aortic Aneurysm, Abdominal/ (572)
2	Aortic Aneurysm, Thoracic/ (89)
3	Iliac Aneurysm/ (14)
4	exp Carotid Artery Diseases/ (1156)
5	Diabetic Foot/ (979)
6	exp Lymphedema/ (564)
7	Mesenteric Ischemia/ (5)
8	Peripheral Arterial Disease/ (1073)
9	exp Varicose Veins/ (1161)
10	exp Venous Insufficiency/ (580)
11	venous disease*.tw. (350)
12	((aort* or artery) adj3 aneurysm*).tw. (1944)
13	iliac aneurysm*.tw. (35)
14	popliteal aneurysm*.tw. (8)
15	subclavian aneurysm*.tw. (0)
16	brachial aneurysm*.tw. (0)
17	peripheral artery aneurysm*.tw. (0)
18	aort* rupture*.tw. (42)
19	Loeys-Dietz.tw. (11)
20	((aort* or artery or blood vessel*) adj5 dissection*).tw. (761)
21	endoleak*.tw. (193)
22	perigraft leak*.tw. (1)
23	(carotid adj3 (disease* or disorder* or atherosclero* or arteriopath* or fistula* or injur* or trauma* or thrombos?s or stenos?s or narrowing* or plaque* or ulcer*)).tw. (2864)
24	(diabetic foot or diabetic feet).tw. (2335)
25	foot ulcer*.tw. (1824)
26	lymph?edema*.tw. (1296)
27	(mesenteric adj3 (isch?emia* or insufficienc* or thrombos?s or embolus)).tw. (124)
28	peripheral arter* disease*.tw. (2863)
29	(varicos* or varix or varices or microvaricos*).tw. (3140)
30	(phlebectasi* or vein ectasi*).tw. (1)
31	((venous or stasis) adj3 ulcer*).tw. (1698)
32	venous insufficienc*.tw. (892)
33	1 or 2 or 3 or 4 or 5 or 6 or 7 or 8 or 9 or 10 or 11 or 12 or 13 or 14 or 15 or 16 or 17 or 18 or 19 or 20 or 21 or 22 or 23 or 24 or 25 or 26 or 27 or 28 or 29 or 30 or 31 or 32 (19050)
34	Endarterectomy, Carotid/ (464)
35	Axillofemoral Bypass Grafting/ (1)
36	Amputation/ (421)
37	((vascular or blood vessel or aort* or endovascular or vein) adj3 surg*).tw. (4754)
38	amputation*.tw. (2857)
39	limb salvage*.tw. (325)
40	aneurysmectom*.tw. (42)
41	lower extremity bypass*.tw. (27)

42	infra?inguinal bypass*.tw. (64)
43	(popliteal bypass* or femoro?popliteal bypass*).tw. (167)
44	aortofemoral bypass*.tw. (13)
45	aortobifemoral bypass*.tw. (29)
46	axillo?femoral bypass*.tw. (4)
47	thoracofemoral bypass*.tw. (1)
48	femoro?femoral bypass*.tw. (5)
49	(femorotibial bypass* or tibial bypass*).tw. (8)
50	pedal bypass*.tw. (1)
51	(endarter?ectom* or thromboendarter?ectom*).tw. (1975)
52	vein stripping*.tw. (65)
53	endovenous.tw. (573)
54	embolectom*.tw. (108)
55	(angioplast* or endoluminal repair* or transluminal arterial dilation* or balloon dilation*).tw. (6574)
56	atherectom*.tw. (391)
57	angioscop*.tw. (66)
58	(mechanical thrombolys?s or mechanical clot disruption*).tw. (29)
59	carotid stent*.tw. (391)
60	34 or 35 or 36 or 37 or 38 or 39 or 40 or 41 or 42 or 43 or 44 or 45 or 46 or 47 or 48 or 49 or 50 or 51 or 52 or 53 or 54 or 55 or 56 or 57 or 58 or 59 (16436)
61	33 or 60 (31451)
62	exp Artificial Intelligence/ (1004)
63	((artificial or computational or machine or ambient) adj3 intelligence).tw. (431)
64	AI.tw. (2862)
65	((computer or automated or machine) adj2 (reasoning or inference*)).tw. (9)
66	computer vision system*.tw. (3)
67	(multi?criteria decision* or multiple criteria decision*).tw. (8)
68	machine learning.tw. (1260)
69	transfer learning.tw. (133)
70	deep learning.tw. (430)
71	hierarchical learning.tw. (4)
72	semi?supervised learning.tw. (2)
73	support vector*.tw. (403)
74	(heuristic* or hyperheuristic* or metaheuristic*).tw. (269)
75	expert system*.tw. (169)
76	fuzzy logic.tw. (44)
77	knowledge?base*.tw. (8)
78	natural language processing*.tw. (158)
79	(neural network* or perceptron*).tw. (1253)
80	connectionist model*.tw. (5)
81	(robotics or remote operation* or telerobotic*).tw. (287)
82	segmentation.tw. (1037)
83	big data.tw. (139)
84	(algorithm* adj4 computer*).tw. (556)
85	62 or 63 or 64 or 65 or 66 or 67 or 68 or 69 or 70 or 71 or 72 or 73 or 74 or 75 or 76 or 77 or 78 or 79 or 80 or 81 or 82 or 83 or 84 (8847)
86	61 and 85 (89)

Supplementary References

1. Shum, J. *et al.* Quantitative Assessment of Abdominal Aortic Aneurysm Geometry. *Ann. Biomed. Eng.* **39**, 277–286 (2011).
2. Macía, I., Graña, M., Maiora, J., Paloc, C. & de Blas, M. Detection of type II endoleaks in abdominal aortic aneurysms after endovascular repair. *Comput. Biol. Med.* **41**, 871–880 (2011).
3. Zhang, H., Kheyfets, V. O. & Finol, E. A. Robust infrarenal aortic aneurysm lumen centerline detection for rupture status classification. *Med. Eng. Phys.* **35**, 1358–1367 (2013).
4. García, G., Tapia, A. & De Blas, M. Computer-supported diagnosis for endotension cases in endovascular aortic aneurysm repair evolution. *Comput. Methods Programs Biomed.* **115**, 11–19 (2014).
5. Li, J. *et al.* Decoding the Genomics of Abdominal Aortic Aneurysm. *Cell* **174**, 1361-1372.e10 (2018).
6. Madani, M. *et al.* Machine Learning Detection of Endoleak Following Endovascular Aortic Repair. *Circulation* **140**, A11214–A11214 (2019).
7. Kerut, E. K., To, F., Summers, K. L., Sheahan, C. & Sheahan, M. Statistical and machine learning methodology for abdominal aortic aneurysm prediction from ultrasound screenings. *Echocardiography* **36**, 1989–1996 (2019).
8. Mommers, E. H. H. *et al.* Can Electric Nose Breath Analysis Identify Abdominal Wall Hernia Recurrence and Aortic Aneurysms? A Proof-of-Concept Study. *Surg. Innov.* **27**, 366–372 (2020).
9. Talebi, S. *et al.* Machine learning for endoleak detection after endovascular aortic repair. *Sci. Rep.* **10**, 18343 (2020).
10. McLenon, M. *et al.* Validation of natural language processing to determine the presence and size of abdominal aortic aneurysms in a large integrated health system. *J. Vasc. Surg.* **0**, (2021).
11. Turton, E. P. L., Scott, D. J. A., Delbridge, M., Snowden, S. & Kester, R. C. Ruptured Abdominal Aortic Aneurysm: a Novel Method of Outcome Prediction Using Neural Network Technology. *Eur. J. Vasc. Endovasc. Surg.* **19**, 184–189 (2000).
12. Hadjianastassiou, V. G. *et al.* Informed prognosis [corrected] after abdominal aortic aneurysm repair using predictive modeling techniques [corrected]. *J. Vasc. Surg.* **43**, 467–473 (2006).
13. Lee, K. *et al.* Surface Curvature as a Classifier of Abdominal Aortic Aneurysms: A Comparative Analysis. *Ann. Biomed. Eng.* **41**, 562–576 (2013).

14. Attallah, O. & Ma, X. Bayesian neural network approach for determining the risk of re-intervention after endovascular aortic aneurysm repair. *Proc. Inst. Mech. Eng. [H]* **228**, 857–866 (2014).

15. Wise, E. S., Hocking, K. M. & Brophy, C. M. Prediction of in-hospital mortality after ruptured abdominal aortic aneurysm repair using an artificial neural network. *J. Vasc. Surg.* **62**, 8–15 (2015).

16. Karthikesalingam, A. *et al.* An Artificial Neural Network Stratifies the Risks of Reintervention and Mortality after Endovascular Aneurysm Repair; a Retrospective Observational study. *PLOS ONE* **10**, e0129024 (2015).

17. Monsalve-Torra, A. *et al.* Using machine learning methods for predicting inhospital mortality in patients undergoing open repair of abdominal aortic aneurysm. *J. Biomed. Inform.* **62**, 195–201 (2016).

18. Thompson, P. C. *et al.* Predictive models for mortality after ruptured aortic aneurysm repair do not predict futility and are not useful for clinical decision making. *J. Vasc. Surg.* **64**, 1617–1622 (2016).

19. Attallah, O. *et al.* Using multiple classifiers for predicting the risk of endovascular aortic aneurysm repair re-intervention through hybrid feature selection. *Proc. Inst. Mech. Eng. [H]* **231**, 1048–1063 (2017).

20. Attallah, O. *et al.* Feature selection through validation and un-censoring of endovascular repair survival data for predicting the risk of re-intervention. *BMC Med. Inform. Decis. Mak.* **17**, 115 (2017).

21. Lee, R. *et al.* Applied Machine Learning for the Prediction of Growth of Abdominal Aortic Aneurysm in Humans. *EJVES Short Rep.* **39**, 24–28 (2018).

22. Parikh, S. A. *et al.* Decision Tree Based Classification of Abdominal Aortic Aneurysms Using Geometry Quantification Measures. *Ann. Biomed. Eng.* **46**, 2135–2147 (2018).

23. Canchi, T., Ng, E. Y., Narayanan, S. & Finol, E. A. On the assessment of abdominal aortic aneurysm rupture risk in the Asian population based on geometric attributes. *Proc. Inst. Mech. Eng. [H]* **232**, 922–929 (2018).

24. Meng, L. *et al.* Assessing fluoroquinolone-associated aortic aneurysm and dissection: Data mining of the public version of the FDA adverse event reporting system. *Int. J. Clin. Pract.* **73**, e13331 (2019).

25. Hirata, K. *et al.* Machine Learning to Predict the Rapid Growth of Small Abdominal Aortic Aneurysm. *J. Comput. Assist. Tomogr.* **44**, 37–42 (2020).

26. Ding, N. *et al.* CT texture analysis predicts abdominal aortic aneurysm post-endovascular aortic aneurysm repair progression. *Sci. Rep.* **10**, 12268 (2020).

27. Zhou, C. *et al.* Machine learning for the prediction of acute kidney injury and paraplegia after thoracoabdominal aortic aneurysm repair. *J. Card. Surg.* **35**, 89–99 (2020).

28. Hyer, J. M. *et al.* Can We Improve Prediction of Adverse Surgical Outcomes? Development of a Surgical Complexity Score Using a Novel Machine Learning Technique. *J. Am. Coll. Surg.* **230**, 43-52.e1 (2020).

29. Mei, H., Xu, Y., Wang, J. & Ma, S. Evaluation of Survival Outcomes of Endovascular Versus Open Aortic Repair for Abdominal Aortic Aneurysms with a Big Data Approach. *Entropy* **22**, 1349 (2020).

30. Kordzadeh, A. *et al.* Prediction, pattern recognition and modelling of complications post-endovascular infra renal aneurysm repair by artificial intelligence. *Vascular* **29**, 171–182 (2021).

31. Jalalahmadi, G., Helguera, M. & Linte, C. A. A machine leaning approach for abdominal aortic aneurysm severity assessment using geometric, biomechanical, and patient-specific historical clinical features. in *Medical Imaging 2020: Biomedical Applications in Molecular, Structural, and Functional Imaging* vol. 11317 1131713 (International Society for Optics and Photonics, 2020).

32. Rengarajan, B. *et al.* A Comparative Classification Analysis of Abdominal Aortic Aneurysms by Machine Learning Algorithms. *Ann. Biomed. Eng.* **48**, 1419–1429 (2020).

33. Olabarriaga, S. D., Rouet, J.-M., Fradkin, M., Breeuwer, M. & Niessen, W. J. Segmentation of thrombus in abdominal aortic aneurysms from CTA with nonparametric statistical grey level appearance modeling. *IEEE Trans. Med. Imaging* **24**, 477–485 (2005).

34. Shum, J. *et al.* Semiautomatic vessel wall detection and quantification of wall thickness in computed tomography images of human abdominal aortic aneurysms. *Med. Phys.* **37**, 638–648 (2010).

35. Maiora, J., Papakostas, G. A., Kaburlasos, V. G. & Grana, M. A proposal of Texture Features for interactive CTA Segmentation by Active Learning. *Stud. Health Technol. Inform.* **207**, 311–320 (2014).

36. Maiora, J., Ayerdi, B. & Graña, M. Random forest active learning for AAA thrombus segmentation in computed tomography angiography images. *Neurocomputing* **126**, 71–77 (2014).

37. Jordanski, M., Radovic, M., Milosevic, Z., Filipovic, N. & Obradovic, Z. Machine Learning Approach for Predicting Wall Shear Distribution for Abdominal Aortic Aneurysm and Carotid Bifurcation Models. *IEEE J. Biomed. Health Inform.* **22**, 537–544 (2018).

38. Wang, D. *et al.* Neural network fusion: a novel CT-MR aortic aneurysm image segmentation method. in *Medical Imaging 2018: Image Processing* vol. 10574 1057424 (International Society for Optics and Photonics, 2018).

39. López-Linares, K. *et al.* Fully automatic detection and segmentation of abdominal aortic thrombus in post-operative CTA images using Deep Convolutional Neural Networks. *Med. Image Anal.* **46**, 202–214 (2018).

40. Mohammadi, S., Mohammadi, M., Dehlaghi, V. & Ahmadi, A. Automatic Segmentation, Detection, and Diagnosis of Abdominal Aortic Aneurysm (AAA) Using Convolutional Neural Networks and Hough Circles Algorithm. *Cardiovasc. Eng. Technol.* **10**, 490–499 (2019).

41. Chandrashekar, A. *et al.* A Deep Learning Pipeline to Automate High-Resolution Arterial Segmentation with or without Intravenous Contrast. *Ann. Surg.* (2021) doi:10.1097/SLA.0000000000004595.

42. Fantazzini, A. *et al.* 3D Automatic Segmentation of Aortic Computed Tomography Angiography Combining Multi-View 2D Convolutional Neural Networks. *Cardiovasc. Eng. Technol.* **11**, 576–586 (2020).

43. Caradu, C., Spampinato, B., Vrancianu, A. M., Bérard, X. & Ducasse, E. Fully automatic volume segmentation of infra-renal abdominal aortic aneurysm CT images with deep learning approaches versus physician controlled manual segmentation. *J. Vasc. Surg.* **0**, (2020).

44. He, X., Avril, S. & Lu, J. Estimating aortic thoracic aneurysm rupture risk using tension–strain data in physiological pressure range: an in vitro study. *Biomech. Model. Mechanobiol.* **20**, 683–699 (2021).

45. Harris, R. J. *et al.* Classification of Aortic Dissection and Rupture on Post-contrast CT Images Using a Convolutional Neural Network. *J. Digit. Imaging* **32**, 939–946 (2019).

46. Huo, D., Kou, B., Zhou, Z. & Lv, M. A machine learning model to classify aortic dissection patients in the early diagnosis phase. *Sci. Rep.* **9**, 2701 (2019).

47. Cheng, J., Tian, S., Yu, L., Ma, X. & Xing, Y. A deep learning algorithm using contrast-enhanced computed tomography (CT) images for segmentation and rapid automatic detection of aortic dissection. *Biomed. Signal Process. Control* **62**, 102145 (2020).

48. Liu, L. *et al.* A study of aortic dissection screening method based on multiple machine learning models. *J. Thorac. Dis.* **12**, (2020).

49. Liu, L. *et al.* An early aortic dissection screening model and applied research based on ensemble learning. *Ann. Transl. Med.* **8**, 1578–1578 (2020).

50. Hata, A. *et al.* Deep learning algorithm for detection of aortic dissection on non-contrast-enhanced CT. *Eur. Radiol.* **31**, 1151–1159 (2021).

51. Cao, L. *et al.* Fully automatic segmentation of type B aortic dissection from CTA images enabled by deep learning. *Eur. J. Radiol.* **121**, (2019).

52. Chen, D. *et al.* Multi-stage learning for segmentation of aortic dissections using a prior aortic anatomy simplification. *Med. Image Anal.* **69**, 101931 (2021).

53. Yu, Y. *et al.* A Three-Dimensional Deep Convolutional Neural Network for Automatic Segmentation and Diameter Measurement of Type B Aortic Dissection. *Korean J. Radiol.* **22**, 168–178 (2021).

54. Serhatlioglu, S., Bozgeyik, Z., Ozkan, Y., Hardalac, F. & Güler, I. Neurofuzzy classification of the effect of diabetes mellitus on carotid artery. *J. Med. Syst.* **27**, 457–464 (2003).

55. Christodoulou, C. I., Pattichis, C. S., Pantziaris, M. & Nicolaides, A. Texture-based classification of atherosclerotic carotid plaques. *IEEE Trans. Med. Imaging* **22**, 902–912 (2003).

56. Yýldýrým, H. *et al.* Classification of the frequency of carotid artery stenosis with MLP and RBF neural networks in patients with coroner artery disease. *J. Med. Syst.* **28**, 591–601 (2004).

57. Mofidi, R. *et al.* Prediction of the exact degree of internal carotid artery stenosis using an artificial neural network based on duplex velocity measurements. *Ann. Vasc. Surg.* **19**, 829–837 (2005).

58. Kyriacou, E. *et al.* Multiscale morphological analysis of the atherosclerotic carotid plaque. *Conf. Proc. Annu. Int. Conf. IEEE Eng. Med. Biol. Soc. IEEE Eng. Med. Biol. Soc. Annu. Conf.* **2005**, 1626–1629 (2005).

59. Kyriacou, E. *et al.* Ultrasound imaging in the analysis of carotid plaque morphology for the assessment of stroke. *Stud. Health Technol. Inform.* **113**, 241–275 (2005).

60. Ubeyli, E. D. & Güler, I. Feature extraction from Doppler ultrasound signals for automated diagnostic systems. *Comput. Biol. Med.* **35**, 735–764 (2005).

61. Derya Übeyli, E. & Güler, İ. Adaptive neuro-fuzzy inference systems for analysis of internal carotid arterial Doppler signals. *Comput. Biol. Med.* **35**, 687–702 (2005).

62. Ubeyli, E. D. & Güler, I. Improving medical diagnostic accuracy of ultrasound Doppler signals by combining neural network models. *Comput. Biol. Med.* **35**, 533–554 (2005).

63. Güler, I. & Übeylı, E. D. A mixture of experts network structure for modelling Doppler ultrasound blood flow signals. *Comput. Biol. Med.* **35**, 565–582 (2005).

64. Ubeyli, E. D. & Güler, I. Wavelet-based neural network analysis of internal carotid arterial Doppler signals. *J. Med. Syst.* **30**, 221–229 (2006).

65. Mougiakakou, S. G. R., Golemati, S., Gousias, I., Nicolaides, A. N. & Nikita, K. S. Computer-aided diagnosis of carotid atherosclerosis based on ultrasound image statistics, laws' texture and neural networks. *Ultrasound Med. Biol.* **33**, 26–36 (2007).

66. Ozşen, S., Kara, S., Latifoğlu, F. & Güneş, S. A new supervised classification algorithm in artificial immune systems with its application to carotid artery Doppler signals to diagnose atherosclerosis. *Comput. Methods Programs Biomed.* **88**, 246–255 (2007).

67. Polat, K., Kara, S., Latifoğlu, F. & Güneş, S. Pattern detection of atherosclerosis from carotid artery doppler signals using fuzzy weighted pre-processing and Least Square Support Vector Machine (LSSVM). *Ann. Biomed. Eng.* **35**, 724–732 (2007).

68. Ceylan, M., Ceylan, R., Özbay, Y. & Kara, S. Application of complex discrete wavelet transform in classification of Doppler signals using complex-valued artificial neural network. *Artif. Intell. Med.* **44**, 65–76 (2008).

69. Übeyli, E. D. Probabilistic neural networks employing Lyapunov exponents for analysis of Doppler ultrasound signals. *Comput. Biol. Med.* **38**, 82–89 (2008).

70. Übeyli, E. D. Statistics over features for internal carotid arterial disorders detection. *Comput. Biol. Med.* **38**, 361–371 (2008).

71. Acharya, U. R. *et al.* Atheromatic™: Symptomatic vs. asymptomatic classification of carotid ultrasound plaque using a combination of HOS, DWT texture. in *2011 Annual International Conference of the IEEE Engineering in Medicine and Biology Society* 4489–4492 (2011). doi:10.1109/IEMBS.2011.6091113.

72. Acharya, R. U. *et al.* Symptomatic vs. Asymptomatic Plaque Classification in Carotid Ultrasound. *J. Med. Syst.* **36**, 1861–1871 (2012).

73. Acharya, U. R. *et al.* Atherosclerotic Risk Stratification Strategy for Carotid Arteries Using Texture-Based Features. *Ultrasound Med. Biol.* **38**, 899–915 (2012).

74. Uğuz, H. Detection of Carotid Artery Disease by Using Learning Vector Quantization Neural Network. *J. Med. Syst.* **36**, 533–540 (2012).

75. Acharya, U. *et al.* Computed tomography carotid wall plaque characterization using a combination of discrete wavelet transform and texture features: A pilot study. *Proc. Inst. Mech. Eng. [H]* **227**, 643–654 (2013).

76. Acharya, U. R. *et al.* Atherosclerotic plaque tissue characterization in 2D ultrasound longitudinal carotid scans for automated classification: a paradigm for stroke risk assessment. *Med. Biol. Eng. Comput.* **51**, 513–523 (2013).

77. Gastounioti, A. *et al.* A Novel Computerized Tool to Stratify Risk in Carotid Atherosclerosis Using Kinematic Features of the Arterial Wall. *IEEE J. Biomed. Health Inform.* **19**, 1137–1145 (2015).

78. Bhosale, S. D. *et al.* Serum Proteomic Profiling to Identify Biomarkers of Premature Carotid Atherosclerosis. *Sci. Rep.* **8**, 9209 (2018).

79. Molinari, F., Raghavendra, U., Gudigar, A., Meiburger, K. M. & Rajendra Acharya, U. An efficient data mining framework for the characterization of symptomatic and asymptomatic carotid plaque using bidimensional empirical mode decomposition technique. *Med. Biol. Eng. Comput.* **56**, 1579–1593 (2018).

80. Polak, A. & Polak, J. F. Internal to Common Carotid Artery Peak Systolic Velocity Ratios for Predicting North American Symptomatic Carotid Endarterectomy Trial Stenosis: Derivation/Validation Study Using a Machine Learning Technique. *J. Vasc. Ultrasound* **43**, 182–185 (2019).

81. Saba, L. *et al.* Ultrasound-based carotid stenosis measurement and risk stratification in diabetic cohort: a deep learning paradigm. *Cardiovasc. Diagn. Ther.* **9**, 43961–43461 (2019).

82. Kats, L., Vered, M., Zlotogorski-Hurvitz, A. & Harpaz, I. Atherosclerotic carotid plaque on panoramic radiographs: neural network detection. *Int. J. Comput. Dent.* **22**, 163–169 (2019).

83. Verde, L. & De Pietro, G. A neural network approach to classify carotid disorders from Heart Rate Variability analysis. *Comput. Biol. Med.* **109**, 226–234 (2019).

84. Skandha, S. S. *et al.* 3-D optimized classification and characterization artificial intelligence paradigm for cardiovascular/stroke risk stratification using carotid ultrasound-based delineated plaque: Atheromatic™ 2.0. *Comput. Biol. Med.* **125**, 103958 (2020).

85. Hsu, K.-C. *et al.* Autodetect extracranial and intracranial artery stenosis by machine learning using ultrasound. *Comput. Biol. Med.* **116**, 103569 (2020).

86. Yin, J. *et al.* Detection of Asymptomatic Carotid Artery Stenosis in High-Risk Individuals of Stroke Using a Machine-Learning Algorithm. *Chin. Med. Sci. J.* **35**, 297–305 (2020).

87. Wu, X., Zhao, Y., Radev, D. & Malhotra, A. Identification of patients with carotid stenosis using natural language processing. *Eur. Radiol.* **30**, 4125–4133 (2020).

88. Zhang, R. *et al.* Identification of high-risk carotid plaque with MRI-based radiomics and machine learning. *Eur. Radiol.* **31**, 3116–3126 (2021).

89. Saba, L. *et al.* Ultrasound-based internal carotid artery plaque characterization using deep learning paradigm on a supercomputer: a cardiovascular disease/stroke risk assessment system. *Int. J. Cardiovasc. Imaging* **37**, 1511–1528 (2021).

90. Aleksic, M. *et al.* Implementation of an Artificial Neuronal Network to Predict Shunt Necessity in Carotid Surgery. *Ann. Vasc. Surg.* **22**, 635–642 (2008).

91. Okser, S. *et al.* Genetic Variants and Their Interactions in the Prediction of Increased Pre-Clinical Carotid Atherosclerosis: The Cardiovascular Risk in Young Finns Study. *PLOS Genet.* **6**, e1001146 (2010).

92. Kyriacou, E. *et al.* Prediction of the time period of stroke based on ultrasound image analysis of initially asymptomatic carotid plaques. in *2015 37th Annual International Conference of the IEEE Engineering in Medicine and Biology Society (EMBC)* 334–337 (2015). doi:10.1109/EMBC.2015.7318367.

93. Hu, X. *et al.* Machine learning to predict rapid progression of carotid atherosclerosis in patients with impaired glucose tolerance. *EURASIP J. Bioinforma. Syst. Biol.* **2016**, 14 (2016).

94. Cheng, C.-A. & Chiu, H.-W. An artificial neural network model for the evaluation of carotid artery stenting prognosis using a national-wide database. in *2017 39th Annual International Conference of the IEEE Engineering in Medicine and Biology Society (EMBC)* 2566–2569 (2017). doi:10.1109/EMBC.2017.8037381.

95. Xiao, F. *et al.* Frequency-Dependent Changes of the Resting BOLD Signals Predicts Cognitive Deficits in Asymptomatic Carotid Artery Stenosis. *Front. Neurosci.* **12**, (2018).

96. Jeon, J. P., Kim, C., Oh, B.-D., Kim, S. J. & Kim, Y.-S. Prediction of persistent hemodynamic depression after carotid angioplasty and stenting using artificial neural network model. *Clin. Neurol. Neurosurg.* **164**, 127–131 (2018).

97. Bai, P. *et al.* Risk Factors of Cerebral Infarction and Myocardial Infarction after Carotid Endarterectomy Analyzed by Machine Learning. *Comput. Math. Methods Med.* **2020**, e6217392 (2020).

98. Jamthikar, A. *et al.* Cardiovascular/stroke risk prevention: A new machine learning framework integrating carotid ultrasound image-based phenotypes and its harmonics with conventional risk factors. *Indian Heart J.* **72**, 258–264 (2020).

99. Tan, J. *et al.* A Machine Learning Approach for Predicting Early Phase Postoperative Hypertension in Patients Undergoing Carotid Endarterectomy. *Ann. Vasc. Surg.* **71**, 121–131 (2021).

100. Adame, I. M. *et al.* Automatic segmentation and plaque characterization in atherosclerotic carotid artery MR images. *Magma N. Y. N* **16**, 227–234 (2004).

101. Molinari, F., Gaetano, L., Balestra, G. & Suri, J. S. Role of fuzzy pre-classifier for high performance LI/MA segmentation in B-mode longitudinal carotid ultrasound images. in *2010 Annual International Conference of the IEEE Engineering in Medicine and Biology* 4719–4722 (2010). doi:10.1109/IEMBS.2010.5626390.

102. Molinari, F., Zeng, G. & Suri, J. S. Greedy Technique and Its Validation for Fusion of Two Segmentation Paradigms Leads to an Accurate Intima–Media Thickness Measure in Plaque Carotid Arterial Ultrasound. *J. Vasc. Ultrasound* **34**, 63–73 (2010).

103. Rosati, S., Molinari, F. & Balestra, G. Feature selection applied to ultrasound carotid images segmentation. in *2011 Annual International Conference of the IEEE Engineering in Medicine and Biology Society* 5161–5164 (2011). doi:10.1109/IEMBS.2011.6091278.

104. Santhiyakumari, N., Rajendran, P. & Madheswaran, M. Medical Decision-Making System of Ultrasound Carotid Artery Intima–Media Thickness Using Neural Networks. *J. Digit. Imaging* **24**, 1112–1125 (2011).

105. Molinari, F., Liboni, W., Pantziaris, M. & Suri, J. S. CALSFOAM-completed automated local statistics based first order absolute moment" for carotid wall recognition, segmentation and IMT measurement: validation and benchmarking on a 300 patient database. *Int. Angiol. J. Int. Union Angiol.* **30**, 227–241 (2011).

106. Destrempes, F., Meunier, J., Giroux, M.-F., Soulez, G. & Cloutier, G. Segmentation of Plaques in Sequences of Ultrasonic B-Mode Images of Carotid Arteries Based on Motion Estimation and a Bayesian Model. *IEEE Trans. Biomed. Eng.* **58**, 2202–2211 (2011).

107. Miloš, R., Dejan, P. & Nenad, F. Mining data from CFD simulation for aneurysm and carotid bifurcation models. in *2011 Annual International Conference of the IEEE Engineering in Medicine and Biology Society* 8311–8314 (2011). doi:10.1109/IEMBS.2011.6092049.

108. Rocha, R., Silva, J. & Campilho, A. Automatic segmentation of carotid B-mode images using fuzzy classification. *Med. Biol. Eng. Comput.* **50**, 533–545 (2012).

109. Hassan, M., Chaudhry, A., Khan, A. & Kim, J. Y. Carotid artery image segmentation using modified spatial fuzzy c-means and ensemble clustering. *Comput. Methods Programs Biomed.* **108**, 1261–1276 (2012).

110. Makhijani, M. K., Balu, N., Yamada, K., Yuan, C. & Nayak, K. S. Accelerated 3D MERGE carotid imaging using compressed sensing with a hidden markov tree model. *J. Magn. Reson. Imaging* **36**, 1194–1202 (2012).

111. Molinari, F. *et al.* Automated carotid IMT measurement and its validation in low contrast ultrasound database of 885 patient Indian population epidemiological study: results of AtheroEdge™ Software. *Int. Angiol. J. Int. Union Angiol.* **31**, 42–53 (2012).

112. Chaudhry, A., Hassan, M., Khan, A. & Kim, J. Y. Automatic Active Contour-Based Segmentation and Classification of Carotid Artery Ultrasound Images. *J. Digit. Imaging* **26**, 1071–1081 (2013).

113. Hassan, M., Chaudhry, A., Khan, A. & Iftikhar, M. A. Robust information gain based fuzzy c-means clustering and classification of carotid artery ultrasound images. *Comput. Methods Programs Biomed.* **113**, 593–609 (2014).

114. Engelen, A. van *et al.* Atherosclerotic Plaque Component Segmentation in Combined Carotid MRI and CTA Data Incorporating Class Label Uncertainty. *PLOS ONE* **9**, e94840 (2014).

115. Thornhill, R. E. *et al.* Can Shape Analysis Differentiate Free-floating Internal Carotid Artery Thrombus from Atherosclerotic Plaque in Patients Evaluated with CTA for Stroke or Transient Ischemic Attack? *Acad. Radiol.* **21**, 345–354 (2014).

116. Rocha, R., Silva, J. & Campilho, A. Automatic detection of the carotid lumen axis in B-mode ultrasound images. *Comput. Methods Programs Biomed.* **115**, 110–118 (2014).

117. Alam, J., Hassan, M., Khan, A. & Chaudhry, A. Robust fuzzy RBF network based image segmentation and intelligent decision making system for carotid artery ultrasound images. *Neurocomputing* **151**, 745–755 (2015).

118. Gao, S. *et al.* Repeatability of in vivo quantification of atherosclerotic carotid artery plaque components by supervised multispectral classification. *Magn. Reson. Mater. Phys. Biol. Med.* **28**, 535–545 (2015).

119. Huang, X.-W. *et al.* Evaluating the risk grade of atherosclerotic plaque using MaZda. **31**, 141–145 (2015).

120. van Engelen, A. *et al.* Multi-Center MRI Carotid Plaque Component Segmentation Using Feature Normalization and Transfer Learning. *IEEE Trans. Med. Imaging* **34**, 1294–1305 (2015).

121. Kutbay, U., Hardalaç, F., Akbulut, M., Akaslan, Ü. & Serhatlıoğlu, S. A Computer-Aided Diagnosis System for Measuring Carotid Artery Intima-Media Thickness (IMT) Using Quaternion Vectors. *J. Med. Syst.* **40**, 149 (2016).

122. Pazinato, D. V. *et al.* Pixel-Level Tissue Classification for Ultrasound Images. *IEEE J. Biomed. Health Inform.* **20**, 256–267 (2016).

123. Araki, T. *et al.* Stroke Risk Stratification and its Validation using Ultrasonic Echolucent Carotid Wall Plaque Morphology: A Machine Learning Paradigm. *Comput. Biol. Med.* **80**, 77–96 (2017).

124. Lekadir, K. *et al.* A Convolutional Neural Network for Automatic Characterization of Plaque Composition in Carotid Ultrasound. *IEEE J. Biomed. Health Inform.* **21**, 48–55 (2017).

125. Biswas, M. *et al.* Deep learning strategy for accurate carotid intima-media thickness measurement: An ultrasound study on Japanese diabetic cohort. *Comput. Biol. Med.* **98**, 100–117 (2018).

126. Qian, C. & Yang, X. An integrated method for atherosclerotic carotid plaque segmentation in ultrasound image. *Comput. Methods Programs Biomed.* **153**, 19–32 (2018).

127. Hassan, M., Murtza, I., Hira, A., Ali, S. & Kifayat, K. Robust spatial fuzzy GMM based MRI segmentation and carotid artery plaque detection in ultrasound images. *Comput. Methods Programs Biomed.* **175**, 179–192 (2019).

128. Savaş, S., Topaloğlu, N., Kazcı, Ö. & Koşar, P. N. Classification of Carotid Artery Intima Media Thickness Ultrasound Images with Deep Learning. *J. Med. Syst.* **43**, 273 (2019).

129. Wu, J. *et al.* Deep morphology aided diagnosis network for segmentation of carotid artery vessel wall and diagnosis of carotid atherosclerosis on black-blood vessel wall MRI. *Med. Phys.* **46**, 5544–5561 (2019).

130. Zhang, Q. *et al.* Plaque components segmentation in carotid artery on simultaneous non-contrast angiography and intraplaque hemorrhage imaging using machine learning. *Magn. Reson. Imaging* **60**, 93–100 (2019).

131. Roy-Cardinal, M.-H., Destrempes, F., Soulez, G. & Cloutier, G. Assessment of Carotid Artery Plaque Components With Machine Learning Classification Using Homodyned-K Parametric Maps and Elastograms. *IEEE Trans. Ultrason. Ferroelectr. Freq. Control* **66**, 493–504 (2019).

132. Zhou, R., Fenster, A., Xia, Y., Spence, J. D. & Ding, M. Deep learning-based carotid media-adventitia and lumen-intima boundary segmentation from three-dimensional ultrasound images. *Med. Phys.* **46**, 3180–3193 (2019).

133. Tsakanikas, V. D. *et al.* A deep learning oriented method for automated 3D reconstruction of carotid arterial trees from MR imaging. in *2020 42nd Annual International Conference of the IEEE Engineering in Medicine Biology Society (EMBC)* 2408–2411 (2020). doi:10.1109/EMBC44109.2020.9176532.

134. Mi, S. *et al.* Detecting Carotid Intima-Media From Small-Sample Ultrasound Images. in *2020 42nd Annual International Conference of the IEEE Engineering in Medicine Biology Society (EMBC)* 2129–2132 (2020). doi:10.1109/EMBC44109.2020.9176282.

135. Zhou, R. *et al.* A Voxel-Based Fully Convolution Network and Continuous Max-Flow for Carotid Vessel-Wall-Volume Segmentation From 3D Ultrasound Images. *IEEE Trans. Med. Imaging* **39**, 2844–2855 (2020).

136. Vila, M. del M. *et al.* Semantic segmentation with DenseNets for carotid artery ultrasound plaque segmentation and CIMT estimation. *Artif. Intell. Med.* **103**, 101784 (2020).

137. Biswas, M. *et al.* Two-stage artificial intelligence model for jointly measurement of atherosclerotic wall thickness and plaque burden in carotid ultrasound: A screening tool for cardiovascular/stroke risk assessment. *Comput. Biol. Med.* **123**, 103847 (2020).

138. Meshram, N. H., Mitchell, C. C., Wilbrand, S., Dempsey, R. J. & Varghese, T. Deep Learning for Carotid Plaque Segmentation using a Dilated U-Net Architecture. *Ultrason. Imaging* **42**, 221–230 (2020).

139. He, C. *et al.* Atherosclerotic Plaque Tissue Characterization: An OCT-Based Machine Learning Algorithm With ex vivo Validation. *Front. Bioeng. Biotechnol.* **8**, (2020).

140. Zhou, R. *et al.* Deep learning-based measurement of total plaque area in B-mode ultrasound images. *IEEE J. Biomed. Health Inform.* 1–1 (2021) doi:10.1109/JBHI.2021.3060163.

141. Zhou, T., Tan, T., Pan, X., Tang, H. & Li, J. Fully automatic deep learning trained on limited data for carotid artery segmentation from large image volumes. *Quant. Imaging Med. Surg.* **11**, 673–683 (2021).

142. Zhao, C., Li, D., Feng, C. & Li, S. OF-UMRN: Uncertainty-guided multitask regression network aided by optical flow for fully automated comprehensive analysis of carotid artery. *Med. Image Anal.* **70**, 101982 (2021).

143. Acharya, R. *et al.* Automated identification of diabetic type 2 subjects with and without neuropathy using wavelet transform on pedobarograph. *J. Med. Syst.* **32**, 21–29 (2008).

144. Acharya, U. R. *et al.* Computer-Based Identification of Type 2 Diabetic Subjects with and Without Neuropathy Using Dynamic Planter Pressure and Principal Component Analysis. *J. Med. Syst.* **36**, 2483–2491 (2012).

145. Li, Z. *et al.* Convolutional Neural Network Based Clustering and Manifold Learning Method for Diabetic Plantar Pressure Imaging Dataset. *J. Med. Imaging Health Inform.* **7**, 639–652 (2017).

146. Huang, Y.-K., Chang, C.-C., Lin, P.-X. & Lin, B.-S. Quantitative Evaluation of Rehabilitation Effect on Peripheral Circulation of Diabetic Foot. *IEEE J. Biomed. Health Inform.* **22**, 1019–1025 (2018).

147. Han, A. & Zhang, Y. Application of refinements on Faster-RCNN in automatic screening of diabetic foot wagner grades. *Acta Medica Mediterr.* 661–665 (2020) doi:10.19193/0393-6384_2020_1_104.

148. Goyal, M. *et al.* Recognition of ischaemia and infection in diabetic foot ulcers: Dataset and techniques. *Comput. Biol. Med.* **117**, 103616 (2020).

149. Singh, K., Singh, V. K., Agrawal, N. K., Gupta, S. K. & Singh, K. Association of Toll-Like Receptor 4 Polymorphisms with Diabetic Foot Ulcers and Application of Artificial Neural Network in DFU Risk Assessment in Type 2 Diabetes Patients. *BioMed Res. Int.* **2013**, e318686 (2013).

150. Lopez-de-Andres, A. *et al.* Predictors of in-hospital mortality following major lower extremity amputations in type 2 diabetic patients using artificial neural networks. *BMC Med. Res. Methodol.* **16**, (2016).

151. Nguyen, H. *et al.* Machine learning models for synthesizing actionable care decisions on lower extremity wounds. *Smart Health* **18**, 100139 (2020).

152. Lin, C. *et al.* The amputation and survival of patients with diabetic foot based on establishment of prediction model. *Saudi J. Biol. Sci.* **27**, 853–858 (2020).

153. Kim, R. B. *et al.* Utilization of smartphone and tablet camera photographs to predict healing of diabetes-related foot ulcers. *Comput. Biol. Med.* **126**, 104042 (2020).

154. Yang, L., Gabriel, N., Hernandez, I., Winterstein, A. G. & Guo, J. Using machine learning to identify diabetes patients with canagliflozin prescriptions at high-risk of lower extremity amputation using real-world data. *Pharmacoepidemiol. Drug Saf.* **30**, 644–651 (2021).

155. Ravaut, M. *et al.* Predicting adverse outcomes due to diabetes complications with machine learning using administrative health data. *Npj Digit. Med.* **4**, 1–12 (2021).

156. Yavuz, M., Ocak, H., Hetherington, V. J. & Davis, B. L. Prediction of Plantar Shear Stress Distribution by Artificial Intelligence Methods. *J. Biomech. Eng.* **131**, (2009).

157. Mukherjee, R. *et al.* Automated Tissue Classification Framework for Reproducible Chronic Wound Assessment. *BioMed Res. Int.* **2014**, e851582 (2014).

158. Liu, C., Netten, J. J. van, Baal, J. G. van, Bus, S. A. & Heijden, F. van der. Automatic detection of diabetic foot complications with infrared thermography by asymmetric analysis. *J. Biomed. Opt.* **20**, 026003 (2015).

159. Wang, L., Pedersen, P. C., Agu, E., Strong, D. M. & Tulu, B. Area Determination of Diabetic Foot Ulcer Images Using a Cascaded Two-Stage SVM-Based Classification. *IEEE Trans. Biomed. Eng.* **64**, 2098–2109 (2017).

160. Babu, K. S., Asit, S. & Sukanta, S. Segmentation of Diabetic Wound by Multidimensional Clustering for Quantitative Assessment of Healing Process. *Curr. Med. Imaging* **14**, 71–76 (2018).

161. Cui, C. *et al.* Diabetic Wound Segmentation using Convolutional Neural Networks. in *2019 41st Annual International Conference of the IEEE Engineering in Medicine and Biology Society (EMBC)* 1002–1005 (2019). doi:10.1109/EMBC.2019.8856665.

162. Ohura, N. *et al.* Convolutional neural networks for wound detection: the role of artificial intelligence in wound care. *J. Wound Care* **28**, S13–S24 (2019).

163. Goyal, M., Reeves, N. D., Rajbhandari, S. & Yap, M. H. Robust Methods for Real-Time Diabetic Foot Ulcer Detection and Localization on Mobile Devices. *IEEE J. Biomed. Health Inform.* **23**, 1730–1741 (2019).

164. Wang, C. *et al.* Fully automatic wound segmentation with deep convolutional neural networks. *Sci. Rep.* **10**, 21897 (2020).

165. Cruz-Vega, I., Hernandez-Contreras, D., Peregrina-Barreto, H., Rangel-Magdaleno, J. de J. & Ramirez-Cortes, J. M. Deep Learning Classification for Diabetic Foot Thermograms. *Sensors* **20**, 1762 (2020).

166. Zoppo, G. *et al.* AI technology for remote clinical assessment and monitoring. *J. Wound Care* **29**, 692–706 (2020).

167. Gindi, G. R., Darken, C. J., O'Brien, K. M., Stetz, M. L. & Deckelbaum, L. I. Neural network and conventional classifiers for fluorescence-guided laser angioplasty. *IEEE Trans. Biomed. Eng.* **38**, 246–252 (1991).

168. Karamchandani, S., Dixit, M., Jain, R. & Bhowmick, M. Application of neural networks in the interpretation of impedance cardiovasograms for the diagnoses of peripheral vascular diseases. *Conf. Proc. Annu. Int. Conf. IEEE Eng. Med. Biol. Soc. IEEE Eng. Med. Biol. Soc. Annu. Conf.* **2005**, 7537–7540 (2005).

169. Huang, C.-J. *et al.* Applications of machine learning techniques to a sensor-network-based prosthesis training system. *Appl. Soft Comput.* **11**, 3229–3237 (2011).

170. Watanabe, T., Yoneyama, T., Toribatake, Y. & Hayashi, H. Main disease classification of intermittent claudication via L1-regularized SVM. in *2013 35th Annual International Conference of the IEEE Engineering in Medicine and Biology Society (EMBC)* 6409–6412 (2013). doi:10.1109/EMBC.2013.6611021.

171. Watanabe, T., Yoneyama, T., Hayashi, H. & Toribatake, Y. Identification of the Causative Disease of Intermittent Claudication through Walking Motion Analysis: Feature Analysis and Differentiation. *Sci. World J.* **2014**, e861529 (2014).

172. Li, C.-M. *et al.* Synchronizing chaotification with support vector machine and wolf pack search algorithm for estimation of peripheral vascular occlusion in diabetes mellitus. *Biomed. Signal Process. Control* **9**, 45–55 (2014).

173. LeMoyne, R., Mastroianni, T., Hessel, A. & Nishikawa, K. Implementation of machine learning for classifying prosthesis type through conventional gait analysis. in *2015 37th Annual International Conference of the IEEE Engineering in Medicine and Biology Society (EMBC)* 202–205 (2015). doi:10.1109/EMBC.2015.7318335.

174. Afzal, N. *et al.* Identifying peripheral arterial disease cases using natural language processing of clinical notes. in *2016 IEEE-EMBS International Conference on Biomedical and Health Informatics (BHI)* 126–131 (2016). doi:10.1109/BHI.2016.7455851.

175. Shawen, N. *et al.* Fall Detection in Individuals With Lower Limb Amputations Using Mobile Phones: Machine Learning Enhances Robustness for Real-World Applications. *JMIR MHealth UHealth* **5**, e8201 (2017).

176. McCarthy, C. P. *et al.* A clinical and proteomics approach to predict the presence of obstructive peripheral arterial disease: From the Catheter Sampled Blood Archive in Cardiovascular Diseases (CASABLANCA) Study. *Clin. Cardiol.* **41**, 903–909 (2018).

177. Jana, B., Oswal, K., Mitra, S., Saha, G. & Banerjee, S. Detection of peripheral arterial disease using Doppler spectrogram based expert system for Point-of-Care applications. *Biomed. Signal Process. Control* **54**, 101599 (2019).

178. Kim, S., Hahn, J.-O. & Youn, B. D. Detection and Severity Assessment of Peripheral Occlusive Artery Disease via Deep Learning Analysis of Arterial Pulse Waveforms: Proof-of-Concept and Potential Challenges. *Front. Bioeng. Biotechnol.* **8**, (2020).

179. Weissler, E. H. *et al.* Use of Natural Language Processing to Improve Identification of Patients With Peripheral Artery Disease. *Circ. Cardiovasc. Interv.* **13**, e009447 (2020).

180. Qutrio Baloch, Z., Raza, S. A., Pathak, R., Marone, L. & Ali, A. Machine Learning Confirms Nonlinear Relationship between Severity of Peripheral Arterial Disease, Functional Limitation and Symptom Severity. *Diagnostics* **10**, 515 (2020).

181. Dai, L. *et al.* Deep learning-based classification of lower extremity arterial stenosis in computed tomography angiography. *Eur. J. Radiol.* **136**, (2021).

182. Stolyarov, R., Carney, M. & Herr, H. Accurate Heuristic Terrain Prediction in Powered Lower-Limb Prostheses Using Onboard Sensors. *IEEE Trans. Biomed. Eng.* **68**, 384–392 (2021).

183. Ross, E. G. *et al.* The use of machine learning for the identification of peripheral artery disease and future mortality risk. *J. Vasc. Surg.* **64**, 1515-1522.e3 (2016).

184. Yurtkuran, A., Tok, M. & Emel, E. A Clinical Decision Support System for Femoral Peripheral Arterial Disease Treatment. *Comput. Math. Methods Med.* **2013**, e898041 (2013).

185. Knežević, A. *et al.* Factors that predict walking ability with a prosthesis in lower limb amputees. *Srp. Arh. Celok. Lek.* **144**, 507–513 (2016).

186. Wurdeman, S., Stevens, P. & Campbell, J. H. Classification Tree to Determine the Probability of Functional Ambulation Levels Among Lower Limb Prosthesis Users. *Arch. Phys. Med. Rehabil.* **100**, e50 (2019).

187. Ross, E. G. *et al.* Predicting Future Cardiovascular Events in Patients With Peripheral Artery Disease Using Electronic Health Record Data. *Circ. Cardiovasc. Qual. Outcomes* **12**, e004741 (2019).

188. Keleş, A. D. & Yucesoy, C. A. Development of a neural network based control algorithm for powered ankle prosthesis. *J. Biomech.* **113**, 110087 (2020).

189. Berger, J. S. *et al.* Evaluation of machine learning methodology for the prediction of healthcare resource utilization and healthcare costs in patients with critical limb ischemia—is preventive and personalized approach on the horizon? *EPMA J.* **11**, 53–64 (2020).

190. Chang, B. *et al.* Deep Learning-Based Risk Model for Best Management of Closed Groin Incisions After Vascular Surgery. *J. Surg. Res.* **254**, 408–416 (2020).

191. Perkins, Z. B. *et al.* Predicting the Outcome of Limb Revascularization in Patients With Lower-extremity Arterial Trauma: Development and External Validation of a Supervised Machine-learning Algorithm to Support Surgical Decisions. *Ann. Surg.* **272**, 564–572 (2020).
192. Bolourani, S. *et al.* Cleaning Up the MESS: Can Machine Learning Be Used to Predict Lower Extremity Amputation after Trauma-Associated Arterial Injury? *J. Am. Coll. Surg.* **232**, 102-113.e4 (2021).
193. Kinner, S. *et al.* Peripheral MRA with k-space Segmentation and Blood-Pool Contrast Agent. *Acad. Radiol.* **18**, 113–119 (2011).
194. Mandelias, K. *et al.* Automatic quantitative analysis of in-stent restenosis using FD-OCT in vivo intra-arterial imaging. *Med. Phys.* **40**, 063101 (2013).
195. Zhang, J. L. *et al.* Exercise-induced calf muscle hyperemia: Rapid mapping of magnetic resonance imaging using deep learning approach. *Physiol. Rep.* **8**, e14563 (2020).
196. Hippe, D. S. *et al.* Confidence Weighting for Robust Automated Measurements of Popliteal Vessel Wall Magnetic Resonance Imaging. *Circ. Genomic Precis. Med.* **13**, e002870 (2020).
197. Jaulent, M. C. *et al.* Fuzzy classification of renal artery lesions from angiograms in the context of angioplasty. *Stud. Health Technol. Inform.* **43 Pt B**, 492–496 (1997).
198. Nielsen, M. *et al.* Interpretation of captopril renography using artificial neural networks. *Clin. Physiol. Funct. Imaging* **25**, 293–296 (2005).
199. Chen, T. *et al.* Prediction of cardiovascular outcomes with machine learning techniques: application to the Cardiovascular Outcomes in Renal Atherosclerotic Lesions (CORAL) study. *Int. J. Nephrol. Renov. Dis.* **12**, 49–58 (2019).
200. Lalande, A., Jaulent, M. C., Cherrak, I., Brunotte, F. & Degoulet, P. Quantifying Stenosis in Renal Arteriograms: A Fuzzy Syntactic Analysis. *Methods Inf. Med.* **38**, 207–213 (1999).
201. Wang, X. *et al.* Comparing different venous thromboembolism risk assessment machine learning models in Chinese patients. *J. Eval. Clin. Pract.* **26**, 26–34 (2020).
202. Fukaya, E. *et al.* Clinical and Genetic Determinants of Varicose Veins. *Circulation* **138**, 2869–2880 (2018).
203. Taylor, R. J., Taylor, A. D. & Smyth, J. V. Using an artificial neural network to predict healing times and risk factors for venous leg ulcers. *J. Wound Care* **11**, 101–105 (2002).

204. Franciscis, S. de *et al.* PredyCLU: a prediction system for chronic leg ulcers based on fuzzy logic; part I – exploring the venous side. *Int. Wound J.* **13**, 1349–1353 (2016).

205. Zhao, X. *et al.* Vertebral artery fusiform aneurysm geometry in predicting rupture risk. *R. Soc. Open Sci.* **5**, 180780.

206. Karhade, A. V. *et al.* Development of machine learning and natural language processing algorithms for preoperative prediction and automated identification of intraoperative vascular injury in anterior lumbar spine surgery. *Spine J.* **0**, (2020).

207. Yeih, D.-F., Wang, Y.-S., Huang, Y.-C., Chen, M.-F. & Lu, S.-S. Physiology-based diagnosis algorithm for arteriovenous fistula stenosis detection. in *2014 36th Annual International Conference of the IEEE Engineering in Medicine and Biology Society* 4619–4622 (2014). doi:10.1109/EMBC.2014.6944653.

208. Kistenev, Y. V. *et al.* Application of multiphoton imaging and machine learning to lymphedema tissue analysis. *Biomed. Opt. Express* **10**, 3353–3368 (2019).

209. Lapuerta, P. *et al.* Neural network assessment of perioperative cardiac risk in vascular surgery patients. *Med. Decis. Mak. Int. J. Soc. Med. Decis. Mak.* **18**, 70–75 (1998).

210. Zhao, Y. *et al.* A CNN-based prototype method of unstructured surgical state perception and navigation for an endovascular surgery robot. *Med. Biol. Eng. Comput.* **57**, 1875–1887 (2019).

211. Amato, A. C. M., Santos, R. V. dos, Saucedo, D. Z. & Amato, S. J. de T. A. Machine learning in prediction of individual patient readmissions for elective carotid endarterectomy, aortofemoral bypass/aortic aneurysm repair, and femoral-distal arterial bypass. *SAGE Open Med.* **8**, 2050312120909057 (2020).

212. Zhou, Y.-J. *et al.* Pyramid attention recurrent networks for real-time guidewire segmentation and tracking in intraoperative X-ray fluoroscopy. *Comput. Med. Imaging Graph.* **83**, 101734 (2020).
